# Model predictive control of sun-coupled innovative heat pumps: a comparison of economic and environmental optimizations

**DOI:** 10.12688/openreseurope.14992.1

**Published:** 2023-01-25

**Authors:** Robin Roure, David Chèze, Mathieu Vallée

**Affiliations:** 1 Univ. Grenoble Alpes, CEA, Liten, INES, 7337, Le-Bourget-du-Lac, France

**Keywords:** Model Predictive Control, Costs optimization, Environmental optimization, Hybrid PVT, Heat Pump, Thermal Energy Storage

## Abstract

**Background:** Heating and cooling in buildings represents a significant amount of the energy demand in the EU, but the market penetration of renewable solutions is still marginal. The SunHorizon project aims at proving the viability and benefits of innovative coupling between heat pumps and various advanced solar panels.

**Methods:** This study focuses on the optimal operation strategies of a technological package composed of hybrid photovoltaic thermal (PVT) panels, a gas driven heat pump and a hot water storage tank. In this work, a model predictive control is developed, based on mixed integer linear programming (MILP) optimization. This model uses innovative elements compared to traditional model predictive control (MPC), with environmental indicators for the electricity grid accounting for imports, co-simulation with TRNSYS using the transmission control protocol (TCP) and modelling of inter-seasonal storage for long and short-term decisions.

The usual minimization of costs is compared to two new optimization approaches, which aims to minimize greenhouse gas (GHG) emissions and maximizing renewable use.

**Results and conclusions:** The results of the optimization of costs and GHG emissions show that gains can be found within the variations in time series related to the electricity grid, but the overall operation strategies remain similar. Optimization of renewable share and self-consumption is another path for control strategy, but with less economic and environmental performance.

## 1 Introduction

Heating and cooling (H&C) for buildings represents 32% of the EU energy demand, of which only 13% comes from renewable energies (HeatRoadMap EU, 2017). In order to comply with the targets of the Paris agreement, new technological solutions for H&C in buildings must be implemented, with a reduced environmental impact as well as financial savings compared to conventional solutions.

The SunHorizon project aims at demonstrating such solutions, with innovative and reliable heat pumps (thermal compression, adsorption, reversible) which, properly coupled and managed with advanced solar panels (thermal, photovoltaic [PV], photovoltaic thermal [PVT]), provide H&C to residential and tertiary buildings with lower emissions, energy bills and fossil fuel dependency. Four different technological packages (TPs) are being developed and demonstrated across EU climates (i.e. Germany, Spain, Belgium and Latvia) and building typologies (small and large-scale residential and tertiary buildings).

This paper is focused on the smart control algorithms that are demonstrated in the Sunisi demo site context: a residential house located near Riga in Latvia, equipped with DUALSUN PVT panels, a BOOSTHEAT gas fired thermal compression heat pump and RATIOTHERM thermal storage (TP2 concept). The control tools aim at finding decision-making strategies that guarantee to cover the energy demand while minimizing costs or environmental impact and complying with comfort constraints.

**Figure 1.**
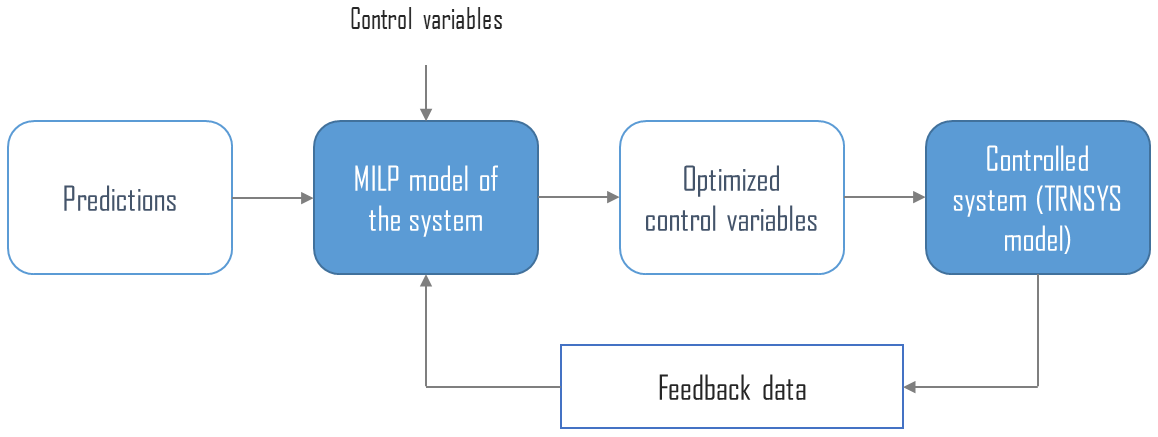
Model predictive control general concept. MILP: mixed integer linear programming.

These decisions making strategies are based on model predictive control (MPC) and mixed integer linear programming (MILP) optimization. MPC is an advanced method of control that uses an optimization algorithm to minimize an objective function while accounting for variable time series such as electricity prices or intermittent solar energy production. The general concept of MPC is presented in Figure 1. 

In the context of innovative energy systems at a district network’s scale, MPC has been proven to offer significant gains compared to rule-based control ^1^ as it can account for variable energy costs and intermittent solar energy production. MILP is an optimization algorithm that is particularly well suited for this type of energy system as it offers good modelling possibilities without increasing the complexity of the resolution too much ^2, 3^. Most of the time this optimization is performed to find the best ways to minimize the operational or capital costs of a new system.

In this paper, we demonstrate the application of MPC with MILP methodology on the TP2 innovative technological package at the residential building scale. This requires developments regarding environmental indicators of the electricity grid, an alternative method to functional mock-up (FMU) interface standards for co-simulation and the modelling of inter-seasonal energy storage.

The main application of MPC is usually for cost minimization. Environmental impacts of the system like GHG emissions and the use of renewable energy are often considered external to the problem and do not represent the focus of the optimization problem. In this paper we propose a comparison of traditional cost minimization with two other control strategies: a GHG emissions minimization strategy and a strategy for the maximization of the use of renewable energy, in order to find what differences in operation these three types of control would induce.

The development of the methods is described in Section 2, and the results are discussed in Section 3. 

## 2 Methods

### 2.1 Test case presentation

This study focuses on the Riga demo site of the SunHorizon project. The building on which the new technological package will be installed is a residential individual house located in Sunisi, near Riga, Latvia. The house was previously equipped with a gas boiler to cover the heating demand.

The installation of 50 m² of DualSun hybrid PVT will be carried out on the premises, for a total installed peak power of 9.6 kWp. A 20kW BoostHeat heat pump will replace the current boiler, the compressor of this heat pump is thermally-driven gas fired, unlike conventional electric vapor compression heat pumps. The building will also be equipped with a RatioTherm hot water storage tank of 1.3m ^3^.

**Figure 2.**
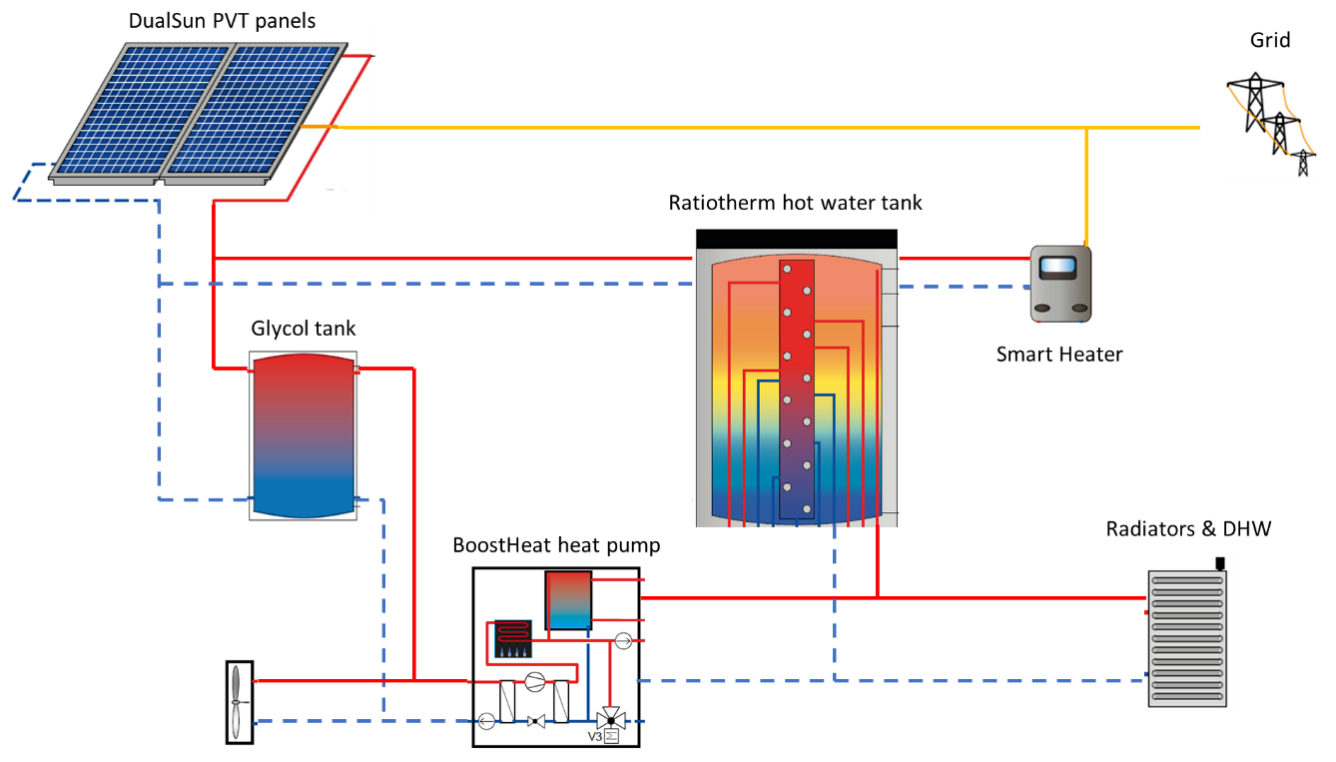
Layout of considered demo site. DHW: domestic hot water; PVT: photovoltaic thermal.

The heat produced by the DualSun panels can be stored in the thermal storage but can also power a secondary loop with a glycol auxiliary storage tank. This glycol loop can be used to increase temperature in the heat pump’s evaporator side compared to external temperature, therefore increasing the seasonal efficiency of the heat pump. A smart heater will also allow the storage of electricity produced by DualSun panels in the water tank. The overall layout of the demo site is presented in Figure 2.

Finally, it is possible in Latvia to benefit from a net metering billing mechanism. Grid net metering is a mechanism that allows the user to feed the PV electricity production into the grid and buy the equivalent amount later for a discounted price. It is a kind of electricity storage on the grid.

The sizing of this innovative technological package was performed by Chèze *et al.* ^4^

### 2.2 MPC implementation

**Figure 3.**
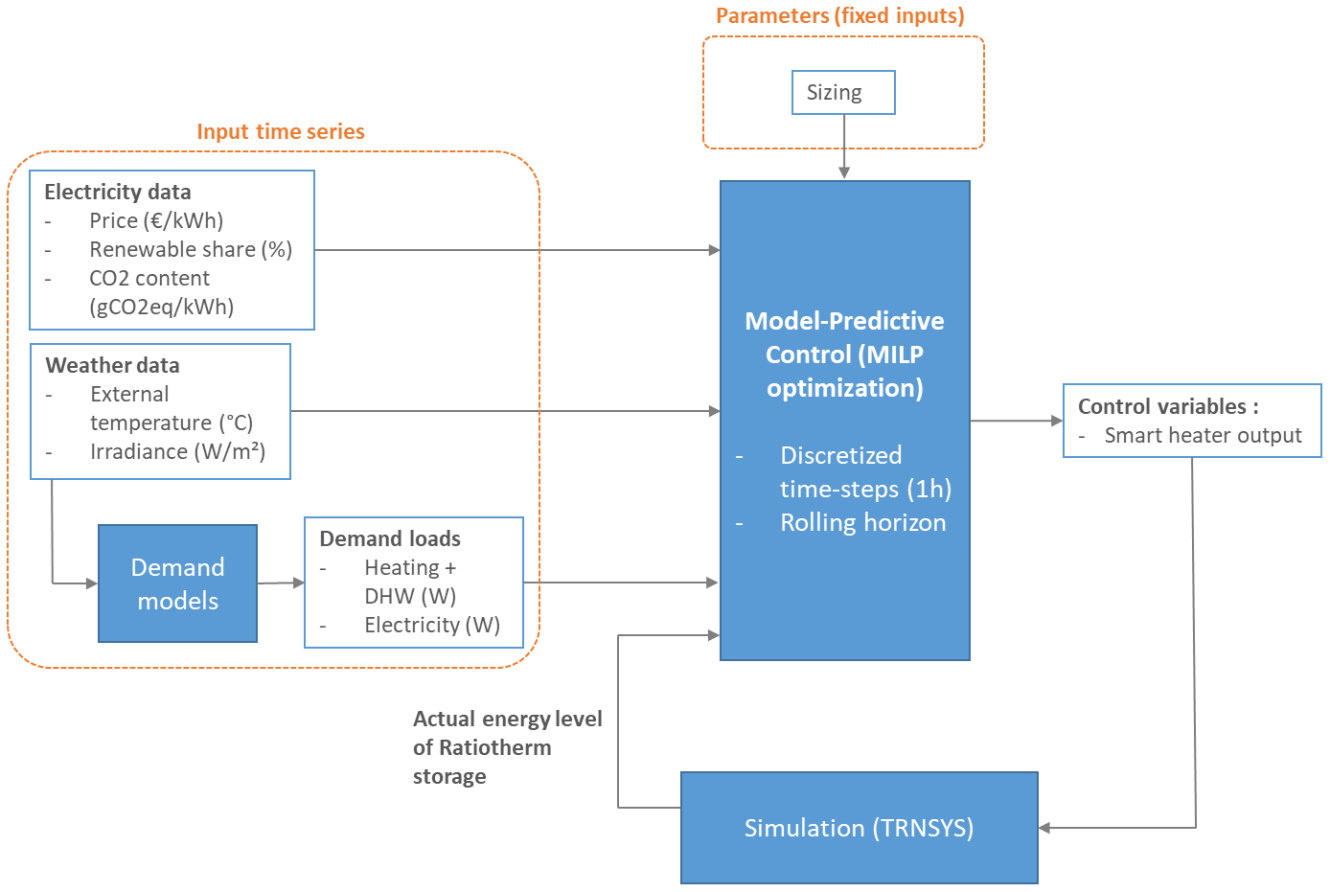
Detailed structure of MPC. DHW: domestic hot water; MILP: mixed integer linear programming; MPC: model predictive control.

MPC is based on MILP optimization. In this type of control, the considered system is modelled as a MILP problem, taking various time series as inputs and calculating the optimized trajectories of a set of control variables in order to minimize an objective function. During the successive optimizations, the controller is given feedback from a TRNSYS digital twin, in order to update the initial state of the MILP problem with actual behavior of the controlled system. The detailed structure of MPC is presented in Figure 3.

The input time series for the MILP model are weather data, heating and electricity dem ands and electricity related data such as variable price and CO2 intensity. Demands are detailed in Section 2.3.1 and electricity indicators in Section 2.3.2.

The MILP model of the considered energy system on which relies the optimization part of the MPC is detailed in Section 2.4.

The optimizer sends to the TRNSYS model optimized control variables, the smart heater power, and gets as feedback from the simulation the actual level of the Ratiotherm storage. The tool implemented to perform this data exchange is described in Section 2.5.

**Figure 4.**
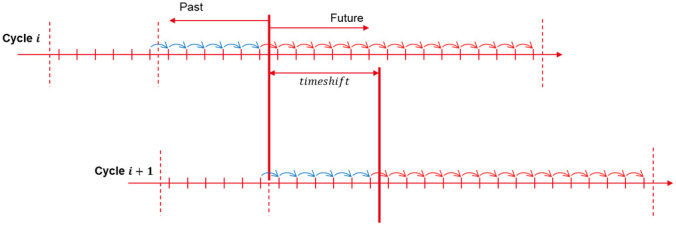
Rolling horizon methodology.

The MPC is using rolling horizon methodology ( Figure 4). For a one-year simulation, the forecasted horizon of each optimization is limited, and optimizations are solved successively with a 1h time-shift between each. The initial state of each optimization is set both by the results of the previous optimization and the feedback from the TRNSYS model.

The grid net metering measure in Riga behaves similarly to seasonal storage. With a typical 48h horizon, the behavior of such storage cannot be forecast. In order to optimize its use, a longer horizon would be required to forecast long-term changes. With a 1h time step and an 8760h horizon, this will induce a high number of constraints that will increase the complexity of the problem and make the computation time skyrocket.

A new methodology is implemented in this paper, proposed by Cuisinier *et al.* ^5^. It uses a horizon with a variable time step, which allows the optimization of long-term decisions as well as short-term decisions.

In addition to the base 48h horizon with a 1h time step, 8 weeks are included in the horizon but with a time step of 168h. Thus, the actual number of time steps on which the optimization needs to be performed is only of 56, but the total period covered by the horizon is 2 months instead of 2 days. With a traditional approach, 1392 time steps would be needed to cover the same period.

### 2.3 Time series development

***2.3.1 Load profiles***. Demand profiles for 2018 were calculated within the project. It uses a detailed building model of the house in Sunisi and weather data measured in Riga in 2018.

Heating load is the aggregated demand of radiators on both floors of the building and of domestic heating water. The electricity load covers the demand of all basic appliances and the use of a chiller in summer (not modelled in our optimization problem as it will not be replaced within the project).

**Figure 5.**
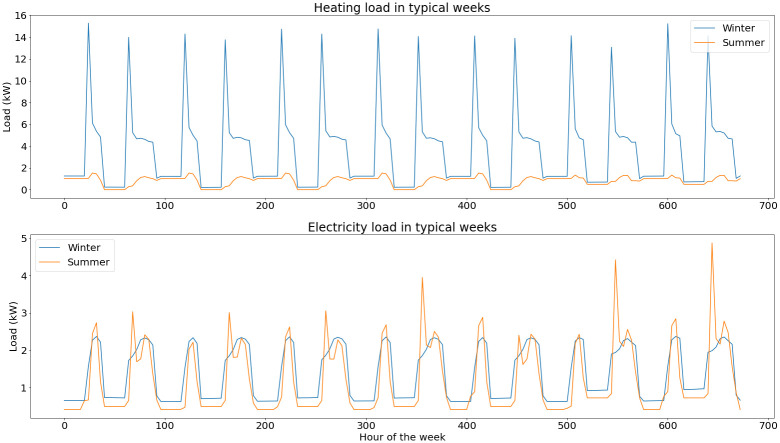
Load profiles for a typical week in winter and in summer.

Typical loads for winter and summer are presented in Figure 5.

***2.3.2 Electricity related data***. In order to optimize the system, external indicators regarding the electricity grid need to be calculated. In addition to the variable electricity costs for costs minimization, indicators such as CO2 intensity and renewable share are needed for environmental impact minimization.

This electricity related data is obtained from the European Network of Transmission System Operators (ENTSOE) platform, where “Augstsprieguma tīkls AS”, the Latvian transmission system operator, shares historical data.

Spot price for the year 2018 in Latvia is used. In addition to these variable costs, a fixed part is added, which corresponds to distribution fees from the TSO (49.3 €/MWh) and subsidies for the development of renewable energies and cogeneration (17.9 €/MWh).

Regarding the environmental indicators, CO2 intensity for generated electricity is calculated using actual generation per production type in Latvia and CO2 intensity factors from the Intergovernmental Panel on Climate Change (IPCC) guidelines ^6^, shown in Table 1.

**Table 1.** CO2 intensity by production technology.

Production technology	CO2 intensity (gCO2/kWh)
Biomass	230
Coal	820
Gas	490
Oil shale	1455
Hydro	24
Nuclear	12
Solar	48
Waste	230
Wind offshore	12
Wind onshore	11
Other	700

**Figure 6.**
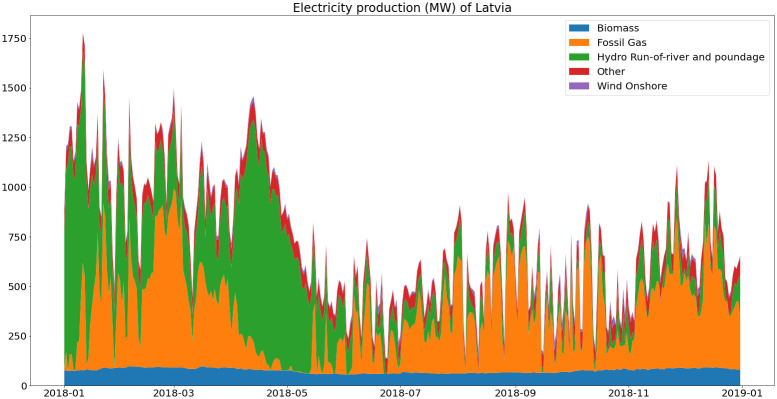
Electricity generation in Latvia (2018).

As shown in Figure 6, base load in Latvia comes mostly from biomass, hydro represents a high share of electricity production but with high seasonal variability and most of the variable load is covered with natural gas.

The mean CO2 intensity of produced electricity in Latvia is therefore around 346 gC02eq/kWh, with 43.7% of the renewable share.

However, when accounting for CO2 emissions of electricity, there are important differences between produced and consumed electricity as mentioned by Tranberg *et al.* ^7^

**Figure 7.**
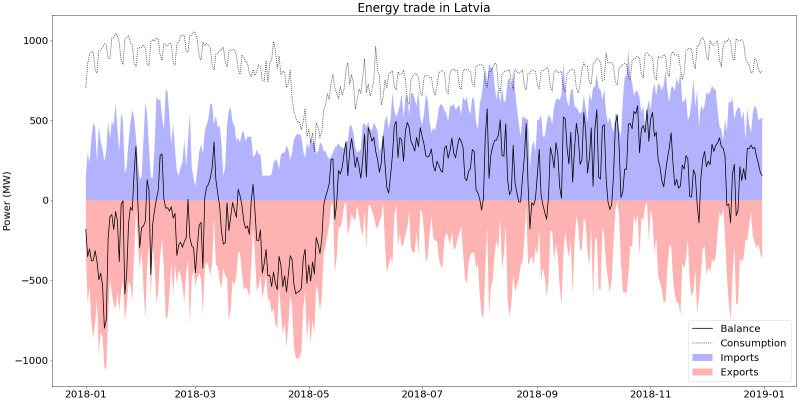
Electricity trade in Latvia (2018).

In Latvia, 11% of the consumed electricity in 2018 came from imports and 68% of these imports came from Estonia (28% from Russia and 3% from Lithuania), as can be seen in Figure 7. Because of this high import share, CO2 intensity for consumed electricity is underestimated if only national electricity generation is considered.

Electricity in Estonia is mainly produced from oil shale, which has a very high CO2 intensity. Oil shale represented 81% of electricity production in 2018, therefore the average CO2 intensity of its electricity production is 1209 gC02eq/kWh).

A new calculation of these indicators is proposed in this paper ( Equation (1)), which accounts more precisely for the part due to imports in the final consumed electricity.


$$\begin{array}{l} \text{For}\;\text{each}\;\text{time}\;t,\\ \left\{ \begin{array}{l} \;\;\;\;\;\;\;\;\;\;\;\;\;\;\;\;\;\;\;\;\;\;\;\;\;\;\;\;\;\;\;\;if\;exports^{t}>imports^{t}:\;CO_{2_{cons}}^{t}=CO_{2_{prod}}^{t}\\ if\;exports^{t}<imports^{t}:\;CO_{2_{cons}}^{t}=\frac{CO_{2_{prod}}^{t}*\;El_{prod}^{t}+\sum_{j\in neighbours}\%imp_{j}^{t}\;*\;Balance^{t}\;*\;CO2_{prod_{j}}^{t}}{El_{prod}^{t}+Balance^{t}} \end{array}\right. \end{array}\;(1)$$

With, for each time *t*, $CO_{2_{cons}}^{t}$ [gCO2eq/kWh] the CO2 intensity of consumed electricity, $CO_{2_{prod}}^{t}$ [gCO2eq/kWh] the CO2 intensity of produced electricity, *exports ^t^* [kWh] the amount of exported energy, *imports ^t^* [kWh] the amount of imported energy, $El_{prod}^{t}$ [kWh] the total of electricity produced in Latvia, $\%imp_{j}^{t}$ the share of imports coming from neighbor *j*, *Balance ^t^* [kWh] the net total of import and $CO_{2_{prod_{j}}}^{t}$ [gCO2eq/kWh] the CO2 intensity of produced electricity from neighbor *j*.

**Figure 8.**
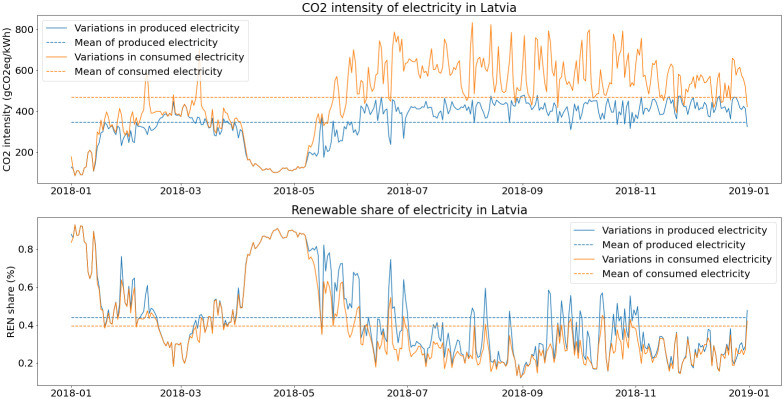
Environmental indicators for the electricity grid in Latvia (2018).

The difference between produced electricity and final consumption is plotted in Figure 8. On average, the final CO2 intensity for the Latvian grid is 468 gCO2eq/kWh, with a renewable share of 39.5 %.

### 2.4 System MILP modelling

The optimization model of the Riga technological package, described in Section 2.1, is based on MILP formalism. The objective of a MILP problem is to find the vector of decision variables *x ^T^* = ( *x*_1_,…, *x _k_*, *x*_*k*+1_,…, *x _n_*) solution of system (2), where *x* is composed of *k* continuous variables and ( *n* – *k*) integer variables.

$$\;\begin{array}{c} \underset{\text{x}}{\min\;}f_{costs}=C^{T}.x\\ with\;\{\begin{array}{c} LHS\leq A.x\leq RHS\\ l_{b}\leq x\leq u_{b} \end{array} \end{array}\;(2)$$

Where *c* [ *n*] is the vector of costs, *A* [ *m* × *n*] is the matrix of linear constraints and *LHS* [ *m*] and *RHS*[ *m*] are the vectors of linear constraints. *l _b_* [ *n*] and *u _b_* [ *n*] are the lower and upper bounds vector of the decision variables, respectively.

The optimization problem was modelled on PERSEE, a modelling software developed internally in CEA (The French Alternative Energies and Atomic Energy Commission) dedicated to optimization and techno-economical assessment of energy systems with multiple energy carriers. PERSEE allows modelling of complex energy system by assembling individual MILP model contributions into a larger problem. The optimization problem is then solved by a CPLEX solver.

**Figure 9.**
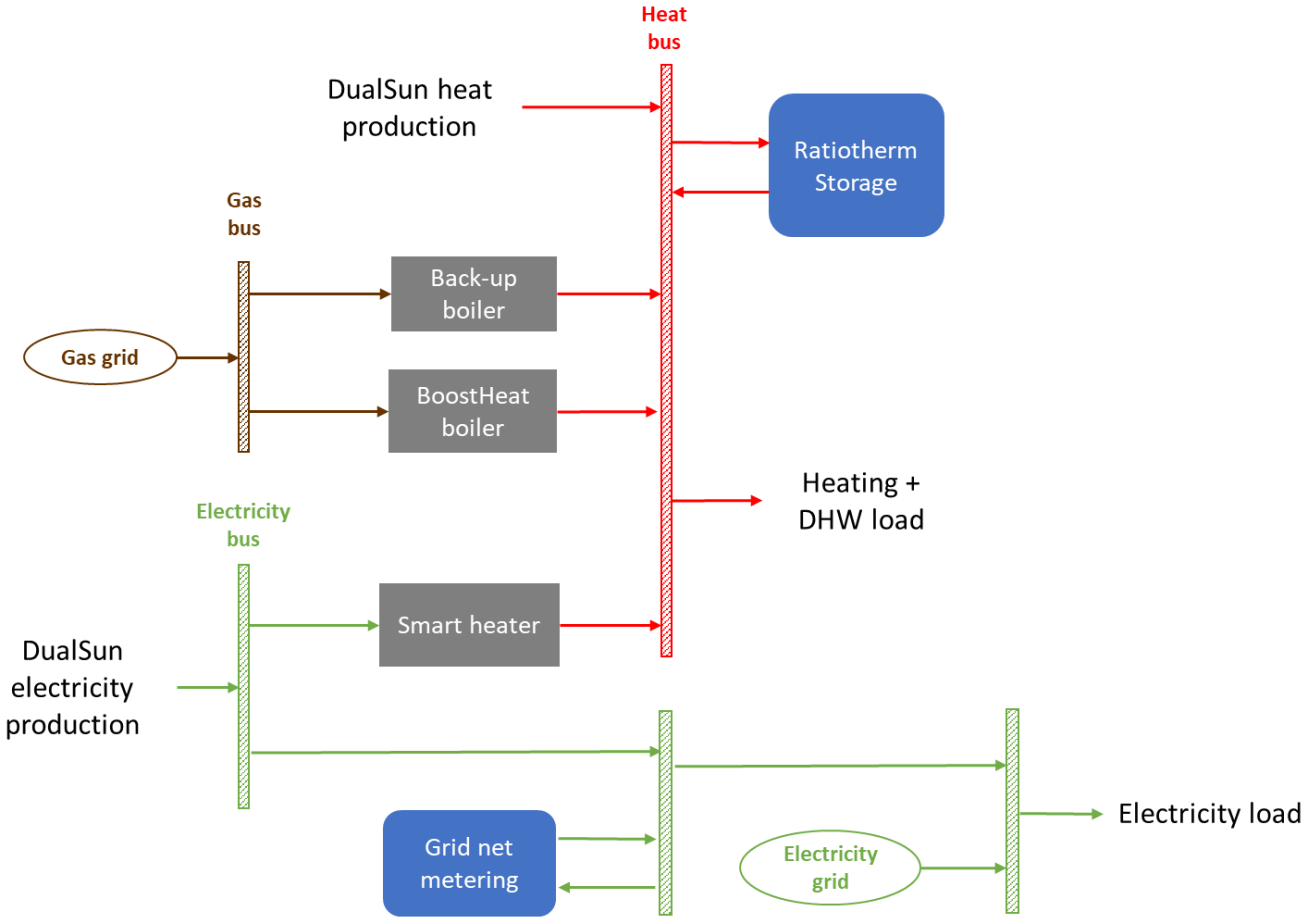
MILP model architecture as implemented in the modelling software. DHW: domestic hot water; MILP: mixed integer linear programming.

Figure 9 gives an overview of the SunHorizon problem architecture as it was implemented inside PERSEE.

The MILP model is based on the following component types:

- Buses: each bus performs a balance of energy flux of its specific energy carrier.
$$\;\sum_{i}P_{in_{i}}^{t}\cdot dt=\sum_{j}P_{out_{j}}^{t}\cdot dt\;(3)$$
  With, for each time *t*, $P_{in_{i}}^{t}$ the i input power to the bus and $P_{out_{j}}^{t}$ the j output powers from the bus
- Energy converters: Smart Heater, BoostHeat heat pump and back-up boiler are energy converters, converting one type of energy carrier to the other with a fixed efficiency.
$$\;P_{out}^{t}=P_{in}^{t}\cdot \eta_{converter}\;(4)$$
  With, for each time *t*, $P_{out}^{t}$ the produced output power of the converter, $P_{in}^{t}$ the input power for the converter and *η _converter_* the efficiency of the converter.
- Storages: Ratiotherm Storage and Grid Net Metering are energy storages that charge and discharge energy to the buses.
$$\;\frac{E_{stored}^{t}-E_{stored}^{t-1}}{dt}=P_{charge}^{t}-P_{discharge}^{t}-K_{loss}\cdot E_{stored}^{t}\;(5)$$
  With, for each time *t*, $E_{stored}^{t}$ the energy stored in the storage, $P_{charge}^{t}$ the charging power of the storage, $P_{discharge}^{t}$ the discharge power of the storage and *K _loss_* an aggregated loss coefficient of the storage.
- Loads and productions: loads and production are imposed time series on a bus.
- Grids: grids offers an infinite source of energy that can be purchased by the system.

In this paper, three objectives functions are compared, one on costs minimization in Equation (6) (referred to as MPC on costs), one on GHG emissions minimization in Equation (7) (referred to as MPC on GHG) and a last one on maximization of renewable energy use in Equation (8) (referred to as MPC on REN).

$$\;f_{obj_{costs}}=\sum_{t}\begin{array}{l} P_{grid}^{t}\cdot Grid_{price}^{t}\cdot dt+Gas_{consumption}^{t}\cdot Gas_{price}\\ \;\;\;\;\;\;+P_{NetMetering}^{t}\cdot NetMetering_{price}\cdot dt \end{array}\;(6)$$

$$\;f_{obj_{GHG}}=\sum_{t}P_{grid}^{t}\cdot Grid_{CO2intensity}^{t}\cdot dt+Gas_{consumption}^{t}\cdot Gas_{CO2intensity}\;(7)$$

$$\;f_{obj_{MPC}}=\sum_{t}P_{grid}^{t}\cdot Grid_{FFshare}^{t}\cdot dt+Gas_{consumption}^{t}\;(8)$$

With, for each time *t*, $P_{grid}^{t}$ [kW] the power extracted from the electricity grid, $Grid_{price}^{t}$ [€/kWh] the instantaneous electricity price, $Gas_{consumption}^{t}$ [kWh] the instantaneous gas consumption of the heat pump, *Gas _price_* [€kWh] the price of gas in Latvia, $P_{NetMetering}^{t}$ [kW] the power drawn from net metering, *NetMetering _price_* [€/kWh] the fixed fee for grid net metering use, $Grid_{CO2intensity}^{t}$ [gCO2eq/kWh] the instantaneous CO2 intensity of electricity from the grid, *Gas _CO2intensity_* [gCO2eq/kWh] the CO2 intensity of natural gas and $Grid_{FFshare}^{t}$ [%] the instantaneous fossil fuel share of electricity from the grid.

### 2.5 Co-simulation with TRNSYS

As part of the MPC methodology, in order to account for the non-linear phenomenon that cannot be modelled through MILP, feedbacks from actual system or simulation model are needed at each timestep to update the state of the system. As real building site is not operational, co-simulation is implemented with the simulation model in TRNSYS. Co-simulation is implemented on PEGASE, a platform developed in CEA ^8^ that provides a framework for the design and deployment of advanced control strategies

Co-simulation is usually done through FMU. Even if an open source project for an FMU tool for TRNSYS is available online, compatibility issues between 32 bits TRNSYS model and 64 bits optimization software made the use of standard FMU impossible.

An alternative method was developed in this paper, using a local TCP (Transmission Control Protocol) server and sockets. TCP is a protocol of the Internet Protocol suite, that provides communication services at a lower level than an application program. It relies on a connection between a server and a client. A module was developed in PEGASE to launch a TCP server and a newly developed TRNSYS type is working as the client side.

In actual operation, both models are running in parallel and at the end of each time step, it pauses until data through the TCP socket is received.

## 3 Results

In this section, results obtained by the MPC for a yearly simulation for the three objective functions are compared ^9^.

**Figure 10.**
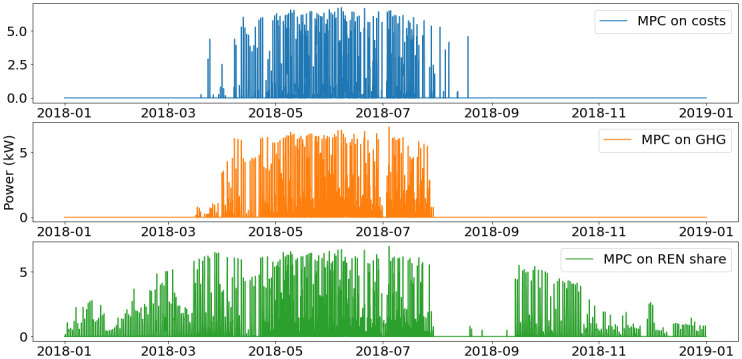
Use of smart heater. GHG: greenhouse gas; MPC: model predictive control; REN: renewable energy use.

Yearly operation of the smart heater and of both storages are presented on Figure 10.

In the MPC on costs and MPC on GHG scenarios, the operation of the smart heater shows similar trends. The smart heater is mostly used when PV production increases from March to July, In November and December, the control is the same as before March, where the smart heater is not used and heat production is covered with the heat pump only.

For the MPC on REN, the smart heater has a more predominant role. It is used as soon as PV electricity is produced. This allows for less use of the heat pump and therefore of natural gas. Consumption from the grid is however increased, as it has a higher renewable share than gas.

**Figure 11.**
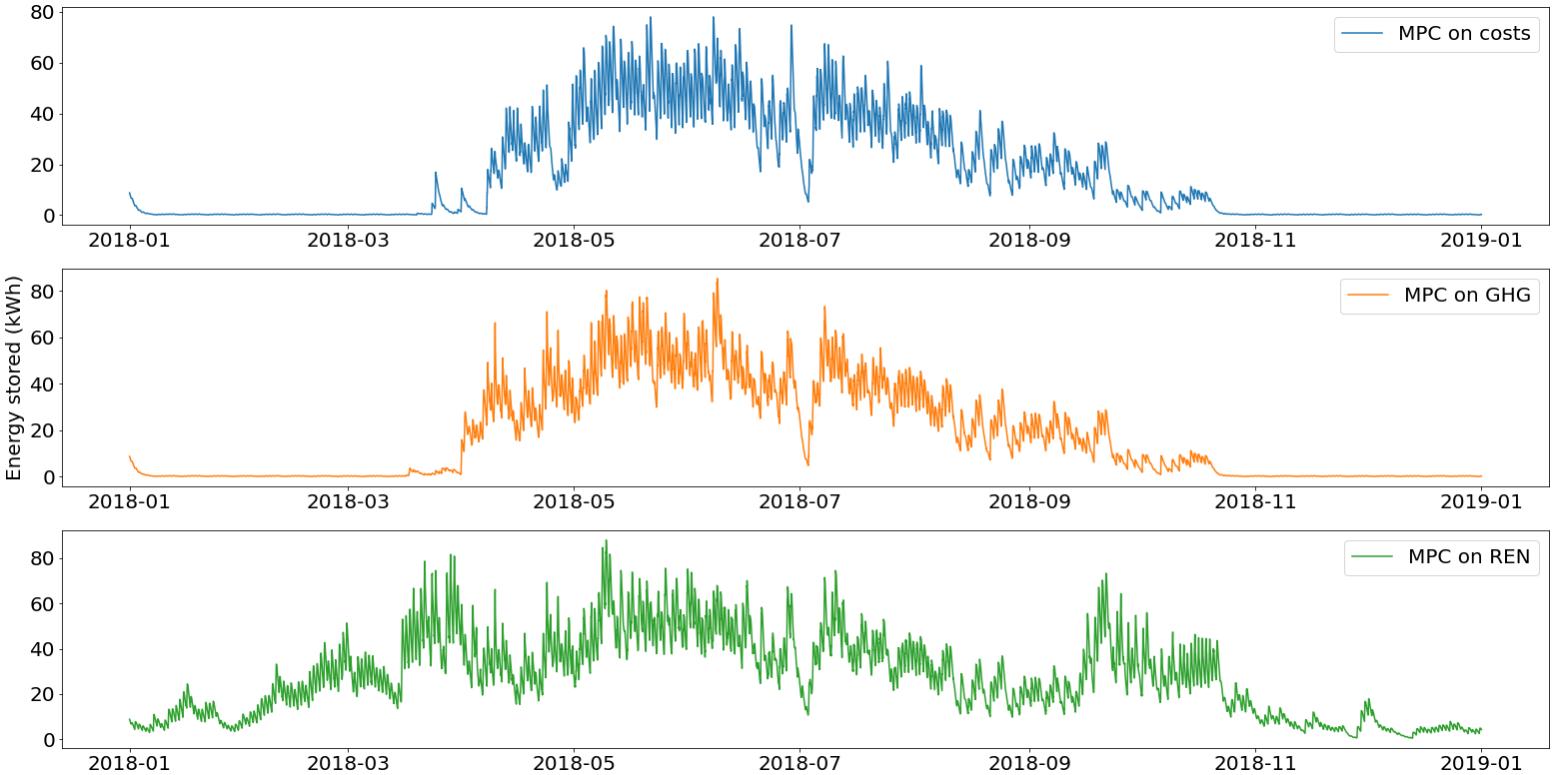
Use of thermal energy storage. GHG: greenhouse gas; MPC: model predictive control; REN: renewable energy use.

**Figure 12.**
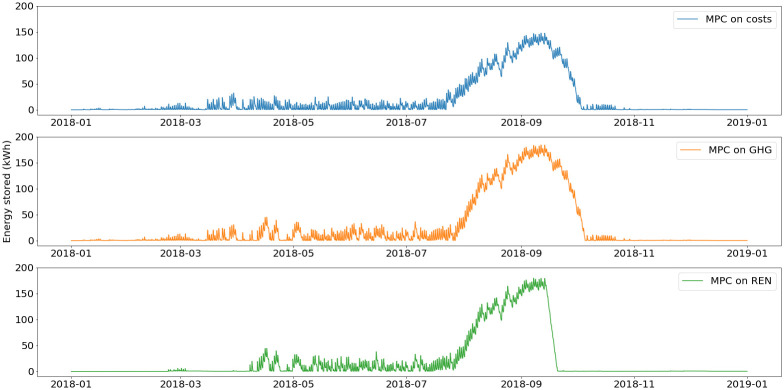
Use of grid metering mechanism. GHG: greenhouse gas; MPC: model predictive control; REN: renewable energy use.

The energy stored in the Ratiotherm storage and the grid net metering are plotted in Figure 11 and Figure 12.

During summer, the excess of solar thermal production is stored in the water tank, which is use as a buffer before the increase of demand in winter. The main differences lie in the energy stored between November and March. With the MPC on REN, because of the high use of the smart heater, the thermal energy storage has higher use during this period. Even when heating demand is high, most of the PV production is converted into heat in order to decrease the use of fossil fuel.

Regarding the net metering, for all scenarios electricity is stored mostly at the end of summer, in order to lower the extraction from grid when PV production decreases. However, because more PV production is converted into heat with the MPC on REN, the cumulative energy stored in net metering is lower.

**Figure 13.**
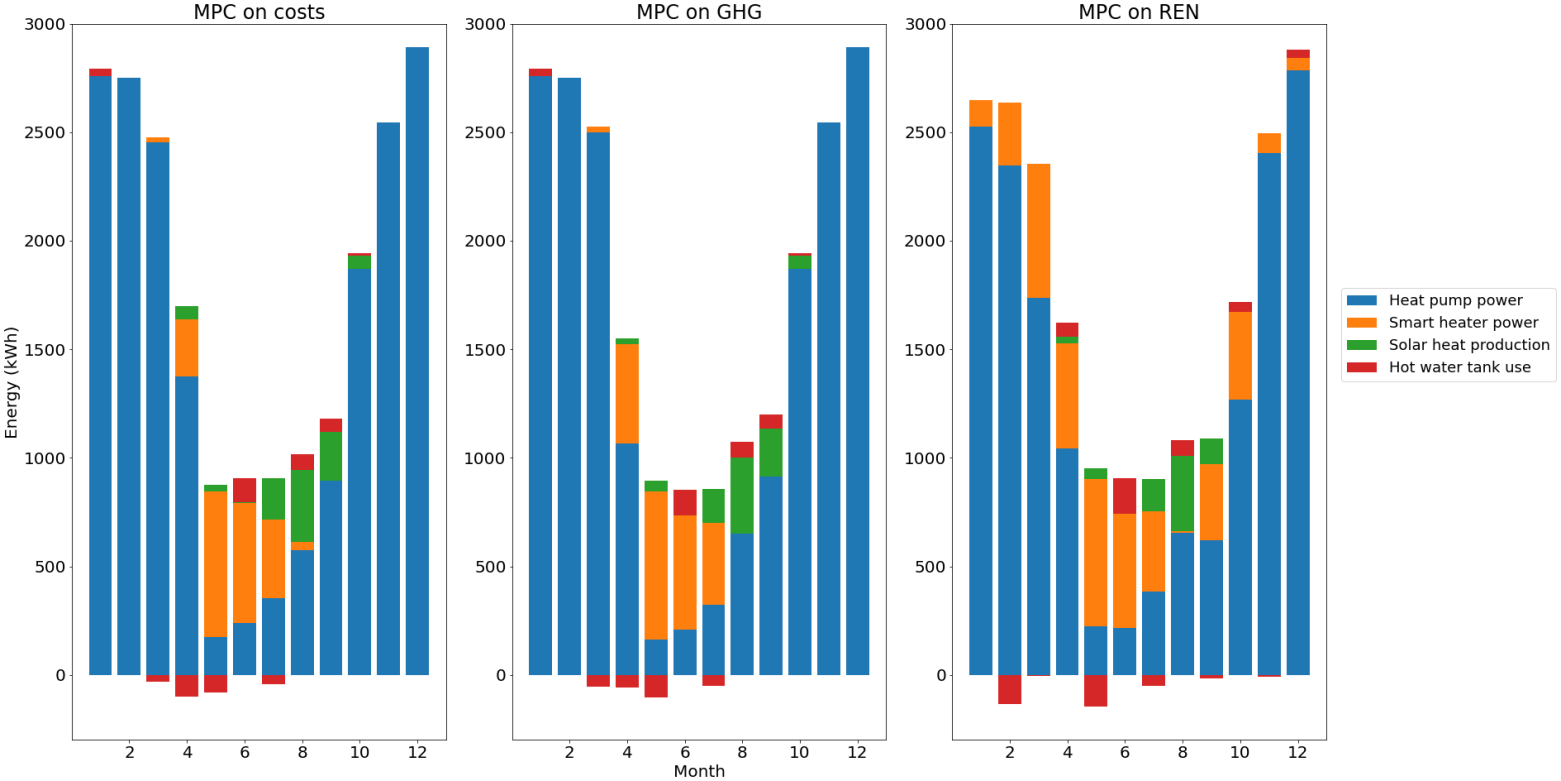
Heat balance per month. GHG: greenhouse gas; MPC: model predictive control; REN: renewable energy use.

**Figure 14.**
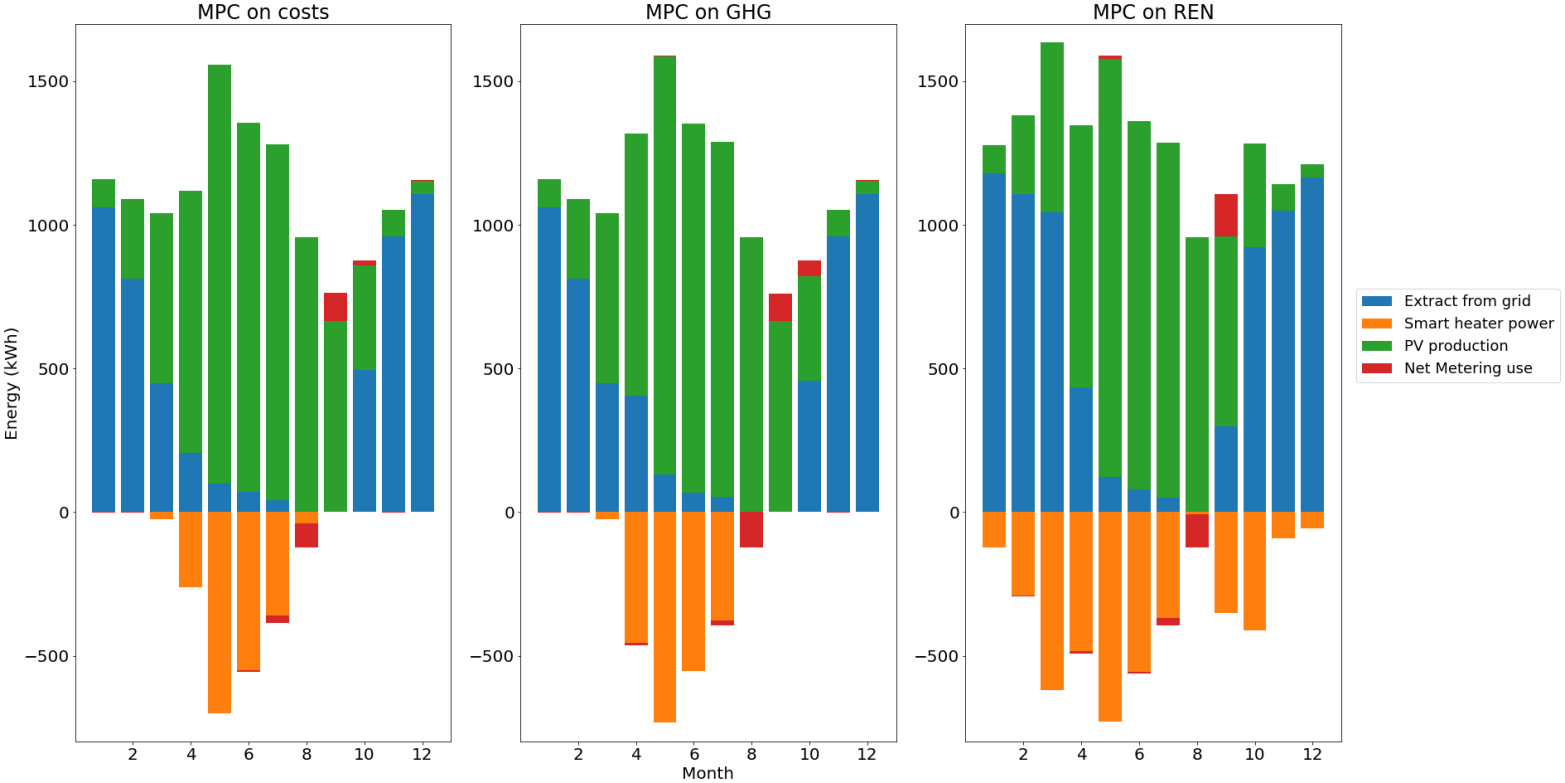
Electricity balance per month. GHG: greenhouse gas; MPC: model predictive control; PV: photovoltaic; REN: renewable energy use.

The total energy balances for heat and electricity are summed up in Figure 13 and Figure 14.

It can be seen in these energy balances that MPC on costs and MPC on GHG have similar behaviors. In all scenarios most of the heat demand is covered by the use of the heat pump. However, as mentioned beforehand, the total heat pump production is lower with the MPC on REN as the smart heater covers some of the demand outside of summer.

For the electricity balance, the impact of the higher use of smart heater shows a lower use of net metering and higher grid consumption with the MPC on REN than the two others.

Main indicators for the two control types can be found in Table 2.

The optimization of costs is 1.5% cheaper than the optimization of GHG, and the emission are almost 1% lower in the second scenario. The two first control types offer gains on either costs or GHG emissions however differences in the final indicators are slight.

This comes from the low level of flexibility of the system, the only control variable being the use of the smart heater. The high CO2 intensity of electricity in Latvia and the low cost of natural gas makes the use of the heat pump an inevitable choice in terms of both costs and GHG emissions.

MPC is however able to profit from the variations in electricity prices or CO2 intensity, to highlight potential gains depending of the chosen control strategy.

The scenario for renewable share offers an increase on both the electricity self-consumption and the renewable energy share. However, this comes with a notable increase in both costs and GHG emissions of the overall system. This control provides interesting results in the case where self-consumption is an important issue, but its economic and environmental interests are low.

**Table 2.** Main indicators for the three control types.

	MPC on costs	MPC on GHG emissions	MPC on REN share
OPEX (€)	1293	1313	1430
GHG emissions (tCO2eq)	6.63	6.59	6.86
Electricity self-consumption (%)	40.3	39.2	46.1
Renewable energy ratio (%)	38.6	39	41.4

GHG: greenhouse gas; MPC: model predictive control; OPEX: operating expenses; PV: photovoltaic; REN: renewable energy

## 4 Conclusion

This paper demonstrates the application of MPC-MILP methodology on an innovative technological package. To proceed with this optimization, the environmental impacts of the electricity grid are calculated, accounting for imports from neighboring countries. Co-simulation is performed outside of the FMU standard by using TCP protocol. Finally, inter-seasonal energy storage can be modelled thanks to an optimization problem with variable time step.

Three control strategies were compared in this paper. Optimization on costs and GHG show that gains can be found within the variations in time series related to the electricity grid, but the overall operation strategies remain similar. Optimization of renewable share and self-consumption shows another path for control strategy but economic and environmental performances are lower.

These control strategies could be improved by performing multi-criteria optimization. Tradeoffs between the three objectives tested in this paper could then be found, but these calculations usually require high computation times. It would also be interesting to test this technological package in other European countries, as lower CO2 intensity in electricity could induce major variations in control between the cost and GHG minimization scenarios.

## Data Availability

Harvard Dataverse: Model Predictive Control of sun-coupled innovative heat pumps: a comparison of economic and environmental optimizations. https://doi.org/10.7910/DVN/3O1RTO ^9^ This project contains the following underlying data: MPC_outputs_costs_scenario.tab MPC_outputs_ghg_scenario.tab MPC_outputs_renshare_scenario.tab Data are available under the terms of the Creative Commons Zero "No rights reserved" data waiver (CC0 1.0 Public domain dedication).

## Software availability

The MPC model was developed on PERSEE and PEGASE, which are proprietary software of CEA. These software programs are used to produce innovative studies for European projects as well as industrial partners. The intellectual property of these programs belongs to CEA, which can sell these studies to companies interested in research and prospective works. Sharing this software cannot be considered as it will deny the added value of CEA in future projects.

Under the ‘obligation to protect results because of legitimate interests or other constraints’ exception of Open Research Europe data policy, we are therefore unable to share the source code associated to this model.

However, in order to replicate the results presented in this article, below is a list of alternative open-source software that can be used as replacements:

- OpenModelica ( https://openmodelica.org/): based on the modeling language Modelica, it offers a free environment to model and analyze complex dynamic systems (alternative to TRNSYS)
- Pyomo ( http://www.pyomo.org/installation) / PulP ( https://pypi.org/project/PuLP/): these Python libraries are used to formulate and solve MILP optimization problems (alternative to PERSEE)
- PyFMI ( https://pypi.org/project/PyFMI/) : this Python library can be used to perform co-simulation with Functional Mock-up Interface standards (alternative to PEGASE)
- GLPK ( https://www.gnu.org/software/glpk/) : is a free MILP solver (alternative to CPLEX)
