# Model predictive control of solar-coupled innovative heat pump: a comparison of economic and environmental optimizations in Latvia

**DOI:** 10.12688/openreseurope.14992.2

**Published:** 2023-05-17

**Authors:** Robin Roure, David Chèze, Mathieu Vallée

**Affiliations:** 1 Univ. Grenoble Alpes, CEA, Liten, INES, 7337, Le-Bourget-du-Lac, France

**Keywords:** Model Predictive Control, Costs optimization, Environmental optimization, Hybrid PVT, Heat Pump, Thermal Energy Storage

## Abstract

**Background:** Heating and cooling in buildings represents a significant amount of the energy demand in the EU, but the market penetration of renewable solutions is still marginal. The SunHorizon project aims at proving the viability and benefits of innovative coupling between heat pumps and various advanced solar panels.

**Methods:** This study focuses on the optimal operation strategies of a technological package located in Latvia, and composed of hybrid photovoltaic thermal (PVT) panels, a gas driven heat pump and a hot water storage tank. In this work, a model predictive control is developed, based on mixed integer linear programming (MILP) optimization. This model uses innovative elements compared to traditional model predictive control (MPC), with environmental indicators for the Latvian electricity grid accounting for imports, co-simulation with TRNSYS using the transmission control protocol (TCP) and modelling of long-term storage for long and short-term decisions.

The usual minimization of costs is compared to two new optimization approaches, which aims to minimize greenhouse gas (GHG) emissions and maximizing renewable use and self-consumption.

**Results and conclusions:** The results of the optimization of costs and GHG emissions show that gains can be found within the variations in time series related to the electricity grid, but the overall operation strategies remain similar. Optimization of renewable share and self-consumption is another path for control strategy, but with less economic and environmental performance.

## 1 Introduction

Heating and cooling (H&C) for buildings represents 32% of the EU energy demand, of which only 13% comes from renewable energies (HeatRoadMap EU, 2017). In order to comply with the targets of the Paris agreement, new technological solutions for H&C in buildings must be implemented, with a reduced environmental impact as well as financial savings compared to conventional solutions.

The SunHorizon project aims at demonstrating such solutions, with innovative and reliable heat pumps (thermal compression, adsorption, reversible) which, properly coupled and managed with advanced solar panels (thermal, photovoltaic [PV], photovoltaic thermal [PVT]), provide H&C to residential and tertiary buildings with lower emissions, energy bills and fossil fuel dependency. Four different technological packages (TPs) are being developed and demonstrated across EU climates (i.e. Germany, Spain, Belgium and Latvia) and building typologies (small and large-scale residential and tertiary buildings).

This paper is focused on the smart control algorithms that are demonstrated in virtual environment in the Sunisi demo site context: a residential house located near Riga in Latvia, equipped with DUALSUN PVT panels, a BOOSTHEAT gas fired thermal compression heat pump and RATIOTHERM thermal storage (TP2 concept). The control tools aim at finding decision-making strategies that guarantee to cover the energy demand while minimizing costs or environmental impact and complying with comfort constraints.

Many works investigate the influence of control algorithms on the actual performance of building energy systems, like the article « Ten questions concerning model predictive control for energy efficient buildings » ^1^ that summarizes well the benefits and issues with MPC for building systems. In many cases, the model-predictive control (MPC) approach makes it possible to reach superior performance, especially thanks to a better anticipation of future conditions and optimized use of energy storage units. As an example, Ghilardi *et al.* ^2^ report up to 80% gains for cooling a building.

**Figure 1.**
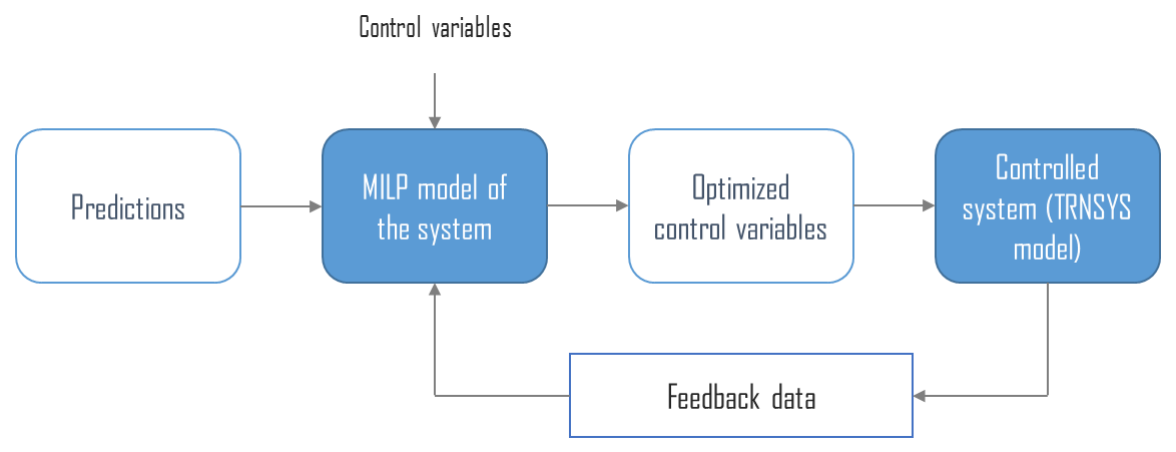
Model predictive control general concept. MILP: mixed integer linear programming.

As illustrated by Figure 1, MPC uses an optimization algorithm to compute optimal control setpoints by minimizing an objective function while accounting for predictions of time series such as electricity prices or intermittent solar energy production. The optimization often relies on the MILP formalism, which is particularly well suited as it offers good modelling capabilities, guarantees optimality and is supported by efficient solvers ^3, 4^.

One of the difficulties for MPC is that significant effort may be required for designing and evaluating the optimization model depending on the controlled systems. Some studies have been performed on similar systems. For instance Herrera *et al.* ^5^ studies a solar absorption cooling system, Chen *et al.* ^6^ studies a heat pump coupled with a PV/T system. In our case, we consider a hybrid system, solar PVT coupled gas fired heat pump and thermal storage, which can provide benefits when the electricity mix has a high carbon content. Hence, the precise setup of the system differs from the previous studies and has different characteristics. In particular, in our case, the large storage tanks allows for storage of thermal energy over several weeks. In order to handle longer optimization horizon in reasonable computational times, previous work ^7^ has proposed a formulation with variable time steps, which we use in this paper to evaluate its performance in another context.

Most of the time the MPC optimization is performed to find the best ways to minimize the operational costs of a system. Environmental impacts of the system like GHG emissions and the use of renewable energy are often considered external to the problem and do not represent the focus of the optimization problem. A key contribution of this paper is to propose a comparison of traditional cost minimization with two other control strategies: a GHG emissions minimization strategy and a strategy for the maximization of the use of renewable energy and self-consumption, in order to find what differences in operation these three types of control would induce. In order to do so, precise environmental indicators of the electricity grid need to be calculated.

As the studied demosite is not installed at the time of this study, the controlled system of the MPC is a non-linear modelling of the technological package, modelled using the software TRNSYS. Due to technical constraints, TRNSYS and the software used for MILP modelling and co-simulation are not using the same computer architecture (32-bits versus 64-bits). An alternative method to traditional functional mock-up (FMU) interface standards needs to be implemented.

In this paper, we therefore demonstrate the application of MPC with MILP methodology on the TP2 innovative technological package at the residential building scale. This requires developments regarding environmental indicators of the electricity grid, an alternative method to functional mock-up (FMU) interface standards for co-simulation and the modelling of long-term energy storage.

The development of the methods is described in Section 2, and the results are discussed in Section 3. 

## 2 Methods

### 2.1 Test case presentation

This study focuses on the Riga demo site of the SunHorizon project where the building on which the new technological package will be installed is a residential individual house located in Sunisi, near Riga, Latvia. The house was previously equipped with a gas boiler to cover the heating demand.

**Figure 2.**
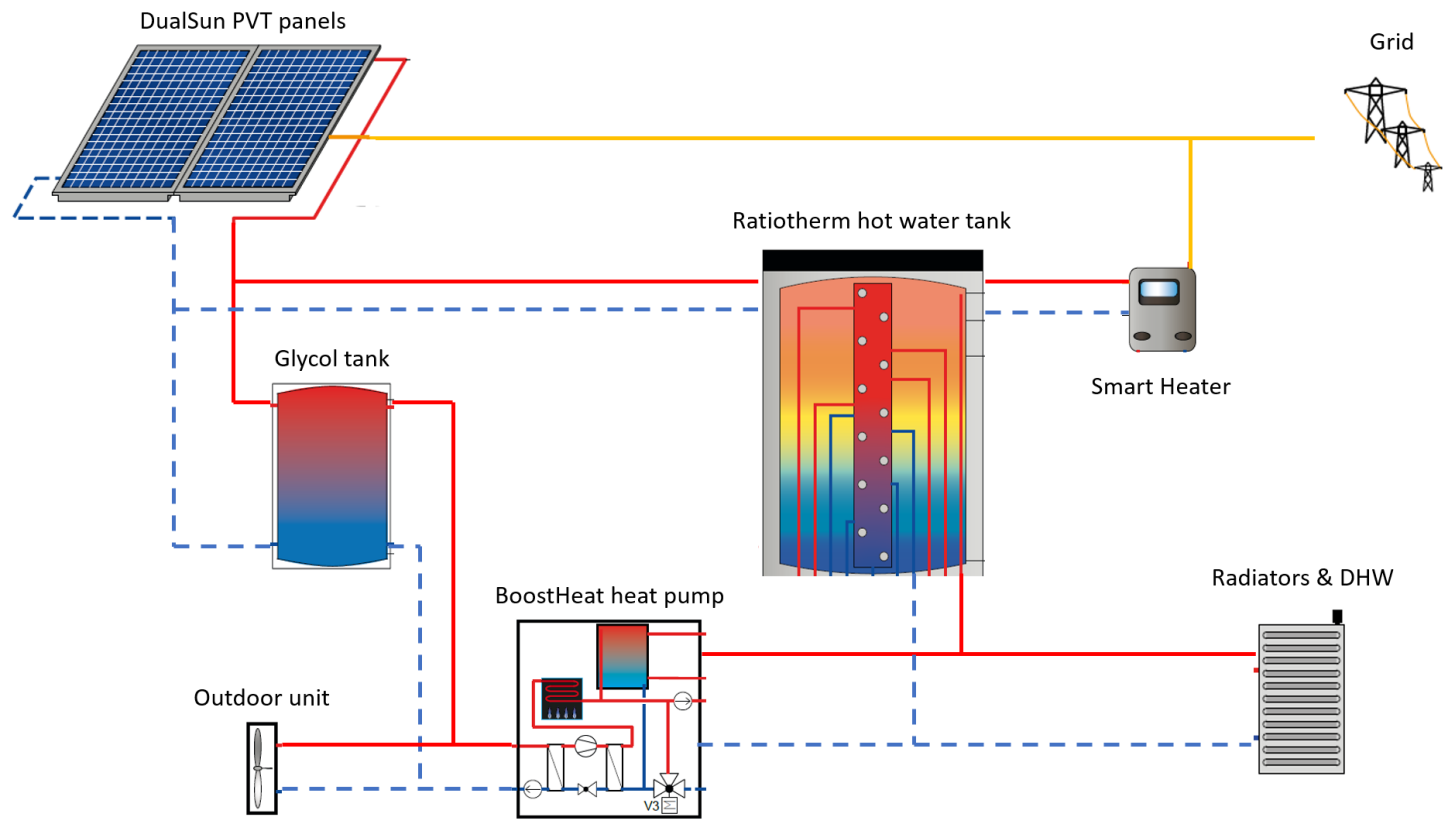
Layout of considered demo site. DHW: domestic hot water; PVT: photovoltaic thermal.

The objective of the project in Riga is to demonstrate the performance of a hybrid system relying on integrated installation of solar, heat pump, thermal storage and controls components as shown in Figure 2, overall energy system layout:

- 50 m² of DualSun solar hybrid PVT uninsulated panels will be carried out on the premises, for a total installed peak power of 9.6 kWp.
- 20kW BoostHeat CO _2_ heat pump in replacement of the current boiler. The indoor unit consists in the brine-to-water heat pump which compressor is thermally driven and gas fired (unlike conventional electric vapor compression heat pumps), for space heating supply, and a secondary gas burner to prepare DHW in compact DHW tank inside the indoor unit. The secondary gas burner is complementing the heat pump’s space heating supply to reach desired temperature flow set point. Details about the thermodynamic working principles of the Boostheat innovative heat pump are given in 8. The lab measurement of Boostheat unit’s Gas Utilization Efficiency (GUE) achieves 2.0 in A7/W35 conditions when connected to the Boostheat outdoor fan coil unit.
- These heat sources components will integrate in the building with hot water storage tank, cold glycol buffer tank and SmartHeater, under the global supervision of controller developed by Ratiotherm technology provider. The SmartHeater component is an electric resistive heater that is switching several resistors in real time to consume up to 15kW. It aims usually at photovoltaic electricity self-consumption objective with regard to the entire building consumption, therefore reducing the amount of electricity fed into the grid as much as can be stored as heat in to the hot water tank, up to 85°C.

The preliminary performance study of the innovative technological package TP2 was performed by SunHorizon partners through TRNSYS ^9^ modelling and simulations together with models of the building, users’ specific electricity and DHW consumptions, Riga climate, and reported in Chèze *et al.* ^10^ together with three other solar and heat pump systems analyzed in SunHorizon project. The main parameter values from TRNSYS components for the current test case TP2 are summarized in Table 1.

**Table 1.** Outlook of the main TRNSYS components from the TP2 test case.

Type Name	Type identifier	Type description
Dualsun PVT Solar collector	Type 816, custom	30 pieces from elementary panel : 1.6 m², 320 Wp ; thermal coefficients following EN ISO 9806 test : a0=55,9% a1= 15,8W/K/m² a2=0W/K²/m²
Hot water tank	Type 340, commercial	1.3 m3, 2 m high, 1.27 W/K loss coefficient
glycol tank	Type 4	0.2 m3, 1.5m high, 1.83 W/K loss coefficient
DHW BH water tank	Type 4	0.065 m3, 50 cm high, 0.74 W/K loss coefficient
Annual specific electricy user consumption profile	Type 9	Annual total consumption of 10.7MWh, calculated from building model in 11
Building	Type 56	Single-family residential house built in 2015, 108 m² living area, 20.2MWh annual heat supply through heating floor and radiators and DHW
BoostHeat unit	Type 5837, custom	Continuous interpolation from the steady state performance tables shared by Boostheat from internal tests of the BH20 unit, for both thermodynamic core and secondary gas burner operation. GUE and electricity consumption are varying according to temperature at the cold (-10 to 20 °C) and hot (30-55°C in Space Heating (SH) operation, 10-85°C in DHW operation) sides of the heat pump, full or part load operation request (25-100% of 20kW-nominal heating capacity in SH operation).

The parametric study revealed a low influence of the storage tanks sizes on the Key Performance Indicators (KPI) like savings of Green House Gas emissions, non-renewable primary Energy consumptions, or cost bills reduction when considering the current electricity Net metering regulation in Latvia. Thus, the decision to incorporate a quite large capacity hot water tank was mainly assuming probable evolution of the renewable electricity feed-in scheme towards self-consumption incentives.

A prototype of this solar and heat pump technology package was built by technology manufacturers in 2021, then tested by CEA following hardware-in-the-loop methodology, so-called Typical Short Sequences (TYPSS). In 12 the overall behavior and control of the real TP2 prototype are compared to previous detailed simulation results. It emphasized the need to set carefully the control parameters of this TP2 to achieve expected energy performance level on such real dynamic system.

Indeed, in order to maximize the solar heat collection efficiency of PVT panels, the reference control strategy in the Riga demo case intends to store solar heat either in the hot water storage or in glycol storage tank according to the coldest tank, which depends on the solar loop temperature grade and heat pump activation rate. The connection of the heat pump’s evaporator to solar heated glycol tank is activated against connection to the outdoor air fan coil, according to the highest temperature level to maximize the current GUE efficiency of the heat pump. After solar thermal heat from PVT panels potentially preheats SH return loop or fresh water in the hot water storage, the Boostheat unit is activated complementary to grant the heat supply to the users at the desired temperature.

In addition, the simulations accounted for the grid net metering mechanism in Latvia. It allows the user to feed into the grid the PV electricity that is not self-consumed by the building and, the following year, to buy the equivalent amount of energy for discounted price where only distribution fees are paid (in average 35% of the actual grid price). It is a kind of electricity storage on the grid.

From the application on TP2 test case detailed TRNSYS simulation, the objective of this work is to analyze the influence on the performance figures of the control decisions relying on MPC upgraded control approach.

### 2.2 MPC implementation

**Figure 3.**
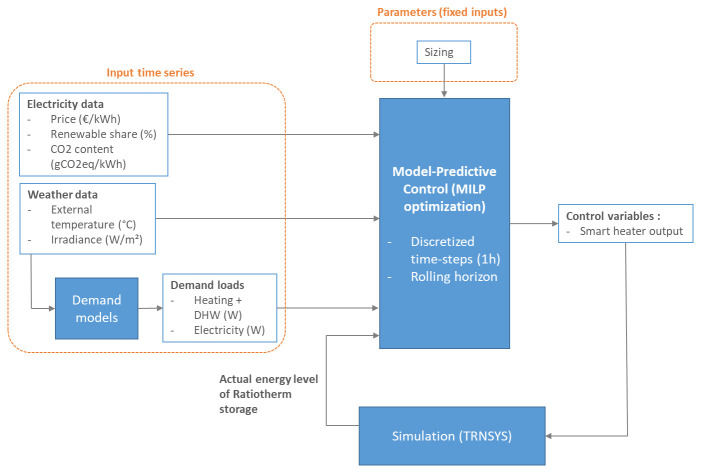
Detailed structure of MPC. DHW: domestic hot water; MILP: mixed integer linear programming; MPC: model predictive control.

MPC is based on MILP optimization. In this type of control, the considered system is modelled as a MILP, taking various time series as inputs and calculating the optimized trajectories of a set of control variables in order to minimize an objective function. During the successive optimizations, the controller is given feedback from a TRNSYS digital twin, in order to update the initial state of the MILP with actual information on the controlled system. The detailed structure of MPC is presented in Figure 3. MILP was chosen for the optimization as it provides computation times that are suitable for future live implementation on demosite.

The input time series for the MILP model are weather data, heating and electricity demand and electricity related data such as variable price and CO2 intensity. Demands are detailed in Section 2.3.1 and electricity indicators in Section 2.3.2.

The MILP model of the considered energy system on which relies the optimization part of the MPC is detailed in Section 2.4.

The optimizer sends to the TRNSYS model optimized control variables, the smart heater power, and gets as feedback from the simulation the actual level of the Ratiotherm storage. Due to technical constraints of the demosite, only the smart heater in the technological package can be controlled by external control algorithms, therefore it represents our main control variable. Other variables could have been implemented in simulation but it was not representative of the actual controlled system. The tool implemented to perform this data exchange is described in Section 2.5.

**Figure 4.**
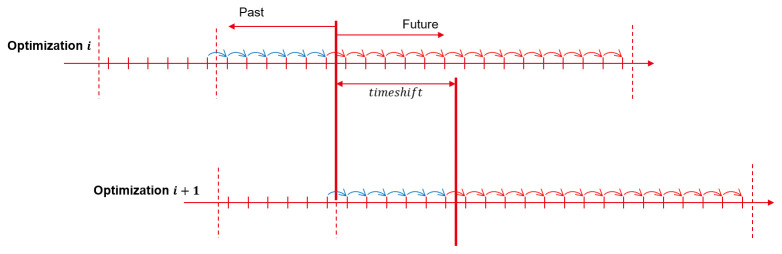
Rolling horizon methodology.

The MPC is using the rolling horizon methodology ( Figure 4). For a one-year simulation, the forecasted horizon of each optimization is limited, and optimizations are solved successively with a 1h time-shift between them. The initial state of each optimization is set both by the results of the previous optimization and the feedback from the TRNSYS model.

The grid net metering measure in Riga behaves similarly to long-term storage. With a typical 48h horizon, the behavior of such storage cannot be forecast, as electricity can be stored in the grid for more than a few days and used later. In order to optimize its use, a longer horizon would be required to forecast long-term changes. With a 1h time step and an 8760h horizon, this will induce a high number of constraints that will increase the complexity of the problem and make the computation time skyrocket.

A new methodology is implemented in this paper, proposed by Cuisinier *et al.* ^7^. It uses a horizon with a variable time step, which allows the optimization of long-term decisions as well as short-term decisions.

**Figure 5.**
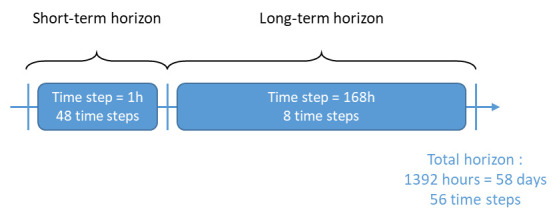
MPC horizon with variable time step.

With this methodology, a control horizon of 58 days (1392 hours) with only 56 time steps is proposed. This global control horizon is the combination of a short-term and a long-term horizon, that have different time steps, as presented in Figure 5. In addition to a short-term 48h horizon (with a 1h time step), a long-term horizon covering 8 weeks is also included (this time with a time step of 168h, hence a week). Thus, the actual number of time steps on which the optimization needs to be performed is only of 56, but the total period covered by the horizon is 2 months and 2 days. With a traditional approach, 1392 time steps would be needed to cover the same period.

### 2.3 Time series development

***2.3.1 Load profiles***. Demand profiles for 2018 were calculated within the project. It uses a detailed building model of the house in Sunisi and weather data measured in Riga in 2018. The details for these loads calculation can be found in 11.

Heating load is the aggregated demand of radiators on both floors of the building and of domestic heating water. The electricity load covers the demand of all basic appliances and the use of a chiller in summer (not modelled in our optimization problem as it will not be replaced within the project).

**Figure 6.**
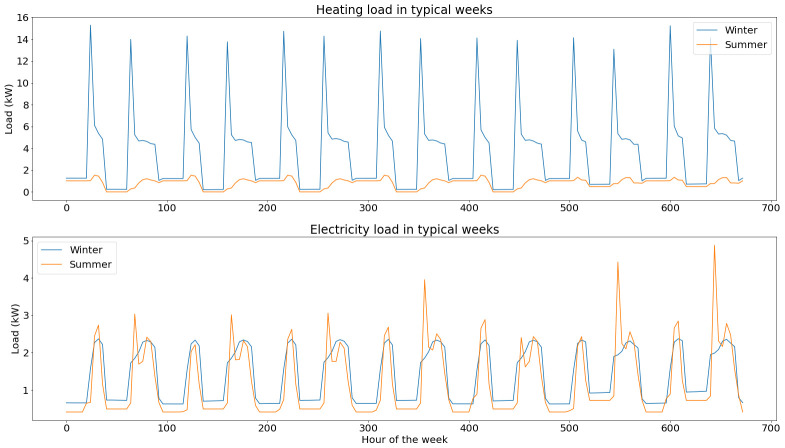
Load profiles for a typical week in winter and in summer.

Typical loads for winter and summer are presented in Figure 6.

***2.3.2 Electricity related data***. In order to optimize the system, external indicators regarding the electricity grid need to be calculated. In addition to the variable electricity costs for costs minimization, indicators such as CO2 intensity and renewable share are needed for environmental impact minimization.

This electricity related data is obtained from the European Network of Transmission System Operators (ENTSOE) platform, where “Augstsprieguma tīkls AS”, the Latvian transmission system operator, shares historical data.

**Figure 7.**
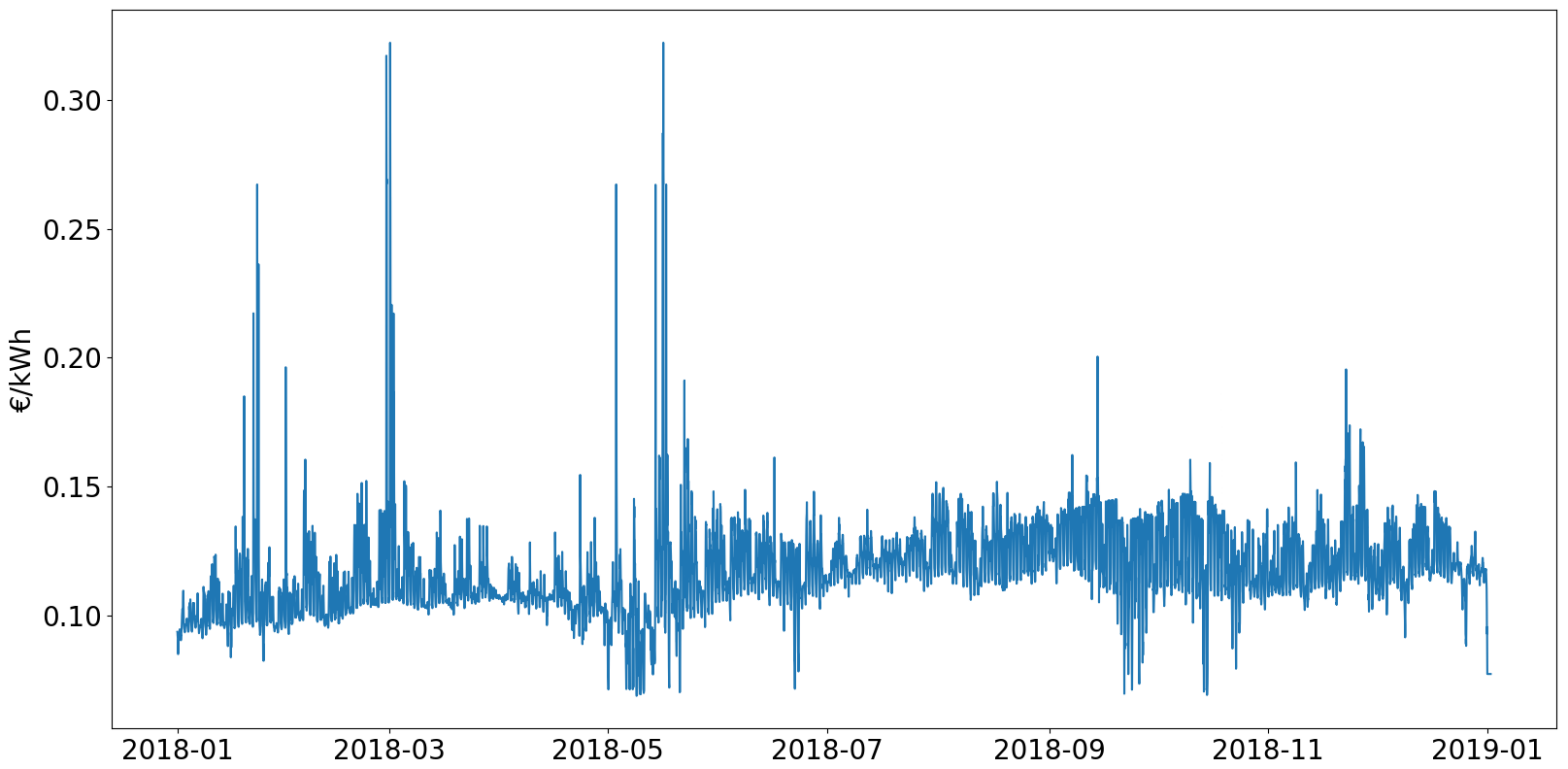
Latvian electricity prices.

Spot prices for the year 2018 in Latvia were used. In addition to these variable costs, a fixed part is added, which corresponds to distribution fees from the TSO (49.3 €/MWh) and subsidies for the development of renewable energies and cogeneration (17.9 €/MWh). The final electricity prices are shown on Figure 7.

Regarding the environmental indicators, CO2 intensity for generated electricity is calculated using actual generation per production type in Latvia and CO2 intensity factors from the Intergovernmental Panel on Climate Change (IPCC) guidelines ^13^, shown in Table 2.

**Table 2.** CO2 intensity by production technology.

Production technology	CO2 intensity (gCO2/kWh)
Biomass	230
Coal	820
Gas	490
Oil shale	1455
Hydro	24
Nuclear	12
Solar	48
Waste	230
Wind offshore	12
Wind onshore	11
Other	700

**Figure 8.**
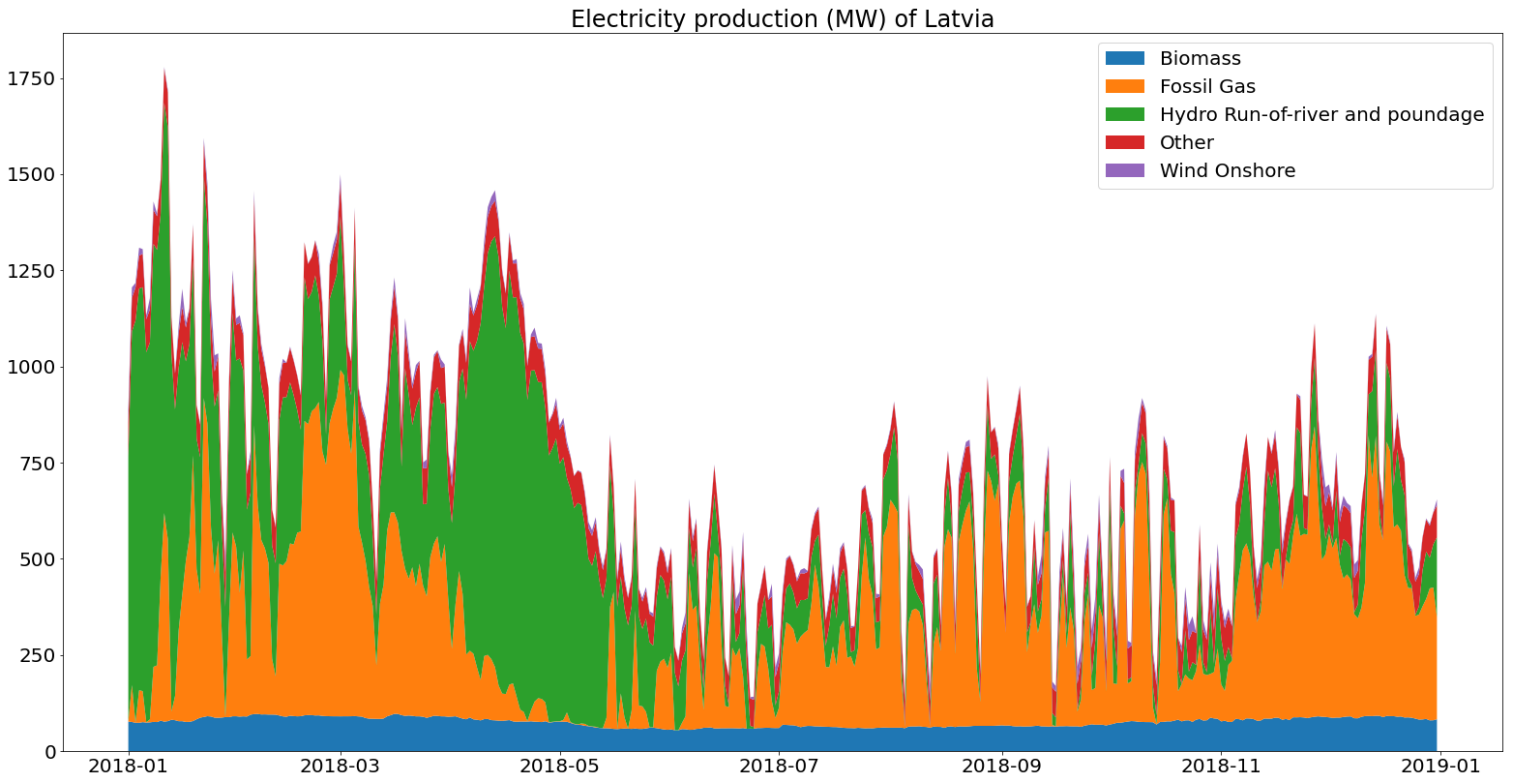
Electricity generation in Latvia (2018).

As shown in Figure 8, base load in Latvia comes mostly from biomass, hydro represents a high share of electricity production but with high seasonal variability and most of the variable load is covered with natural gas.

The mean CO2 intensity of produced electricity in Latvia is therefore around 346 gC02eq/kWh, with 43.7% of the renewable share.

However, when accounting for CO2 emissions of electricity, there are important differences between produced and consumed electricity as mentioned by Tranberg *et al.* ^14^.

**Figure 9.**
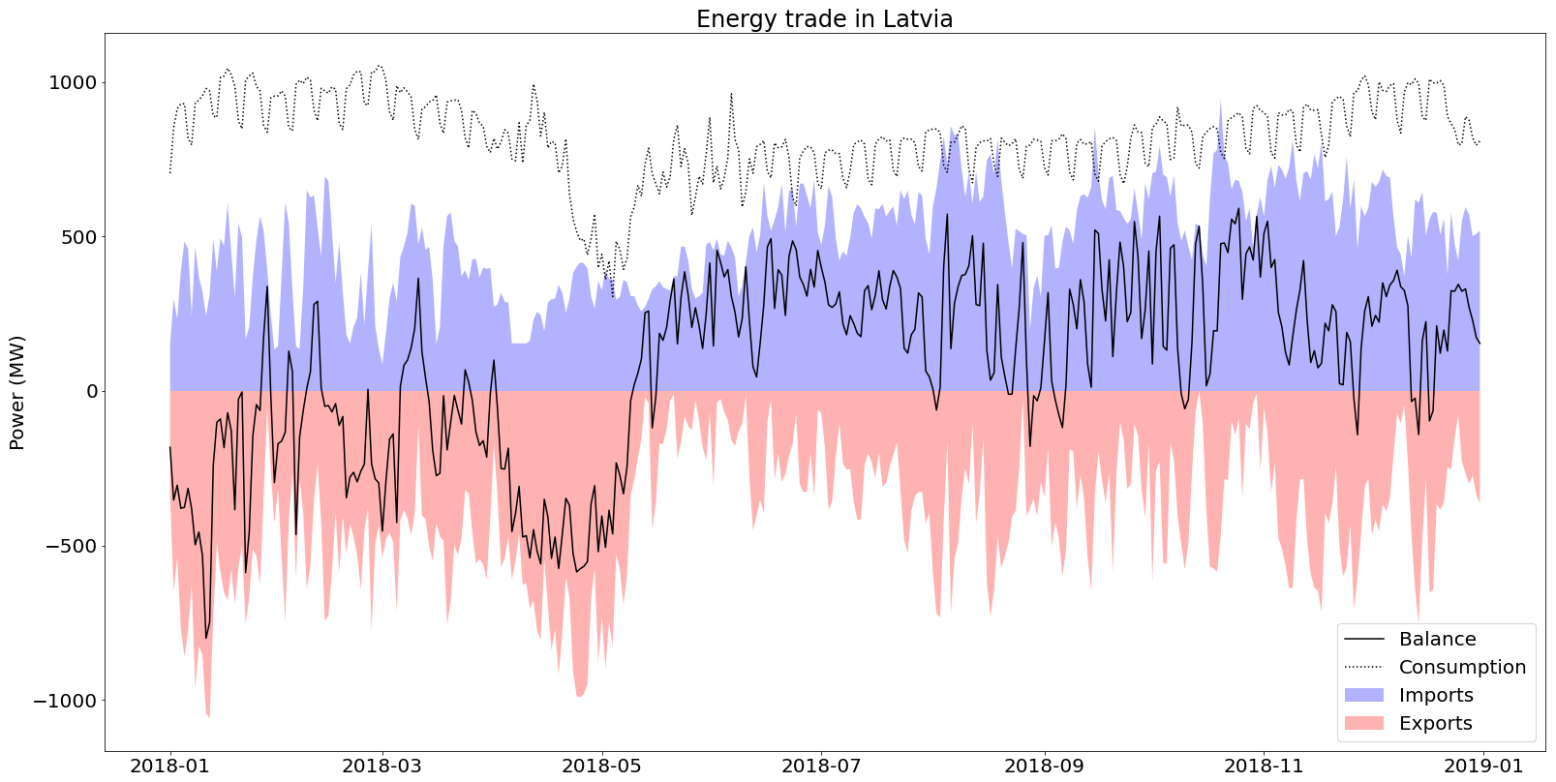
Electricity trade in Latvia (2018).

In Latvia, 11% of the consumed electricity in 2018 came from imports and 68% of these imports came from Estonia (28% from Russia and 3% from Lithuania), as can be seen in Figure 9. Because of this high import share, CO2 intensity for consumed electricity is underestimated if only national electricity generation is considered.

Electricity in Estonia is mainly produced from oil shale, which has a very high CO2 intensity. Oil shale represented 81% of electricity production in 2018, therefore the average CO2 intensity of its electricity production is 1209 gC02eq/kWh).

A new calculation of these indicators is proposed in this paper ( Equation (1)), which accounts more precisely for the part due to imports in the final consumed electricity.


$$\begin{array}{l} \text{For}\;\text{each}\;\text{time}\;t,\\ \left\{ \begin{array}{l} \;\;\;\;\;\;\;\;\;\;\;\;\;\;\;\;\;\;\;\;\;\;\;\;\;\;\;\;\;\;\;\;if\;exports^{t}>imports^{t}:\;CO_{2_{cons}}^{t}=CO_{2_{prod}}^{t}\\ if\;exports^{t}<imports^{t}:\;CO_{2_{cons}}^{t}=\frac{CO_{2_{prod}}^{t}*\;El_{prod}^{t}+\sum_{j\in neighbours}\%imp_{j}^{t}\;*\;Balance^{t}\;*\;CO2_{prod_{j}}^{t}}{El_{prod}^{t}+Balance^{t}} \end{array}\right. \end{array}\;(1)$$

With, for each time *t*, $CO_{2_{cons}}^{t}$ [gCO2eq/kWh] the CO2 intensity of consumed electricity, $CO_{2_{prod}}^{t}$ [gCO2eq/kWh] the CO2 intensity of produced electricity, *exports ^t^* [kWh] the amount of exported energy, *imports ^t^* [kWh] the amount of imported energy, $El_{prod}^{t}$ [kWh] the total of electricity produced in Latvia, $\%imp_{j}^{t}$ the share of imports coming from neighbor *j*, *Balance ^t^* [kWh] the net total of import and $CO_{2_{prod_{j}}}^{t}$ [gCO2eq/kWh] the CO2 intensity of produced electricity from neighbor *j*.

**Figure 10.**
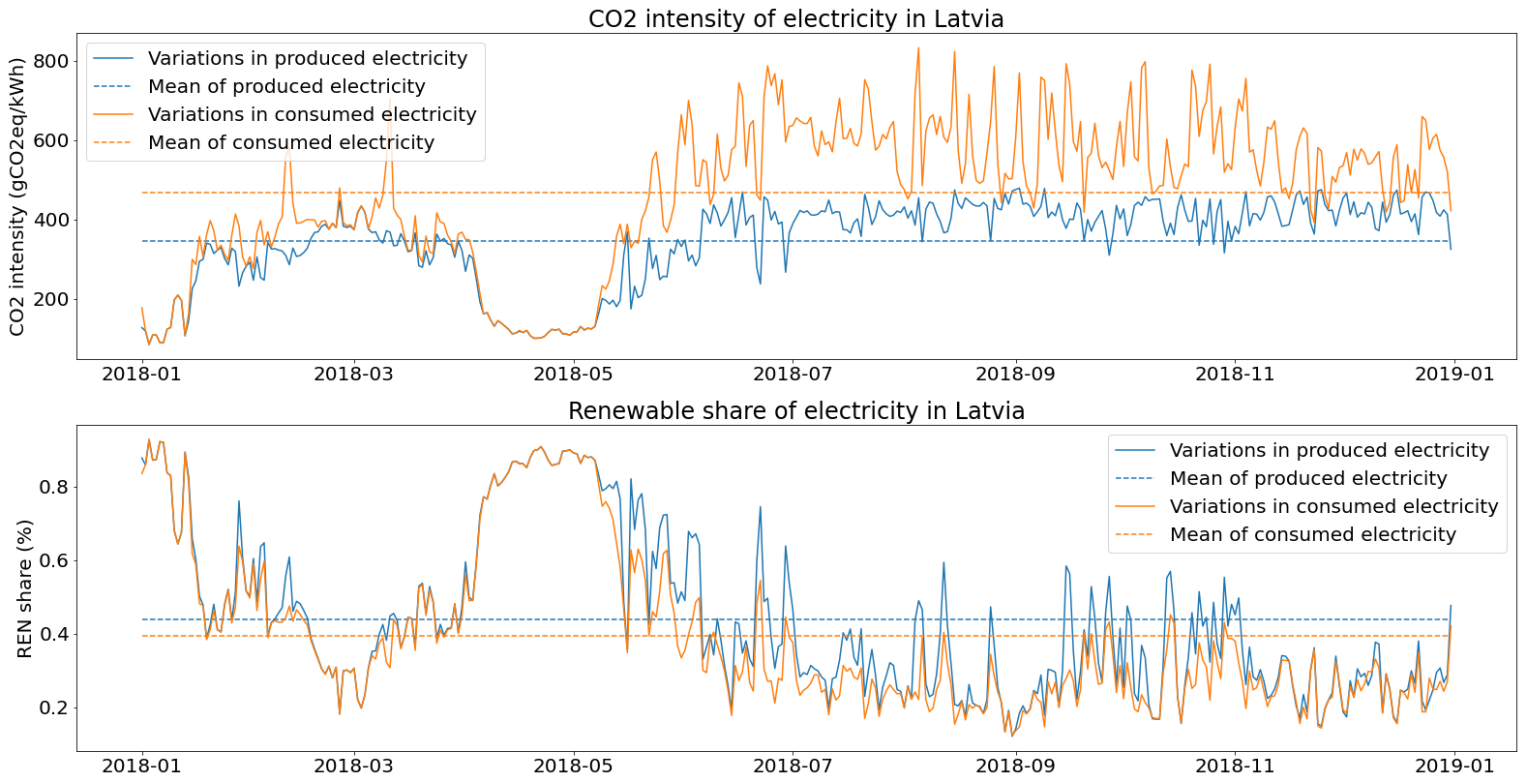
Environmental indicators for the electricity grid in Latvia (2018).

The difference between produced electricity and final consumption is plotted in Figure 10. On average, the final CO2 intensity for the Latvian grid is 468 gCO2eq/kWh, with a renewable share of 39.5 %.

This MPC uses perfect forecast for the above-mentioned electricity and weather time series. In live implementation of the controller, time series forecasting algorithms (with machine learning for example) could be implemented for better accuracy in the results, but it is outside the scope of this study. Prediction of variables such as CO2 intensity of electricity could prove rather complex as it seems to require a great amount of explanatory variables, as well as reliable forecasts for market, power generation and weather data ^15^.

### 2.4 System MILP modelling

The optimization model of the Riga technological package, described in Section 2.1, is based on MILP formalism. The objective of a MILP problem is to find the vector of decision variables *x ^T^* = ( *x*_1_,…, *x _k_*, *x*_*k*+1_,…, *x _n_*) solution of system (2), where *x* is composed of *k* continuous variables and ( *n* – *k*) integer variables.

$$\;\begin{array}{c} \underset{\text{x}}{\min\;}f_{costs}=C^{T}.x\\ with\;\{\begin{array}{c} LHS\leq A.x\leq RHS\\ l_{b}\leq x\leq u_{b} \end{array} \end{array}\;(2)$$

Where *c* [ *n*] is the vector of costs, *A* [ *m* × *n*] is the matrix of linear constraints and *LHS* [ *m*] and *RHS*[ *m*] are the vectors of linear constraints. *l _b_* [ *n*] and *u _b_* [ *n*] are the lower and upper bounds vector of the decision variables, respectively.

The optimization problem was modelled on PERSEE ^16^, a modelling software developed internally in CEA (The French Alternative Energies and Atomic Energy Commission) dedicated to optimization and techno-economical assessment of energy systems with multiple energy carriers. PERSEE allows modelling of complex energy system by assembling individual MILP model contributions into a larger problem. The optimization problem is then solved by a CPLEX solver.

**Figure 11.**
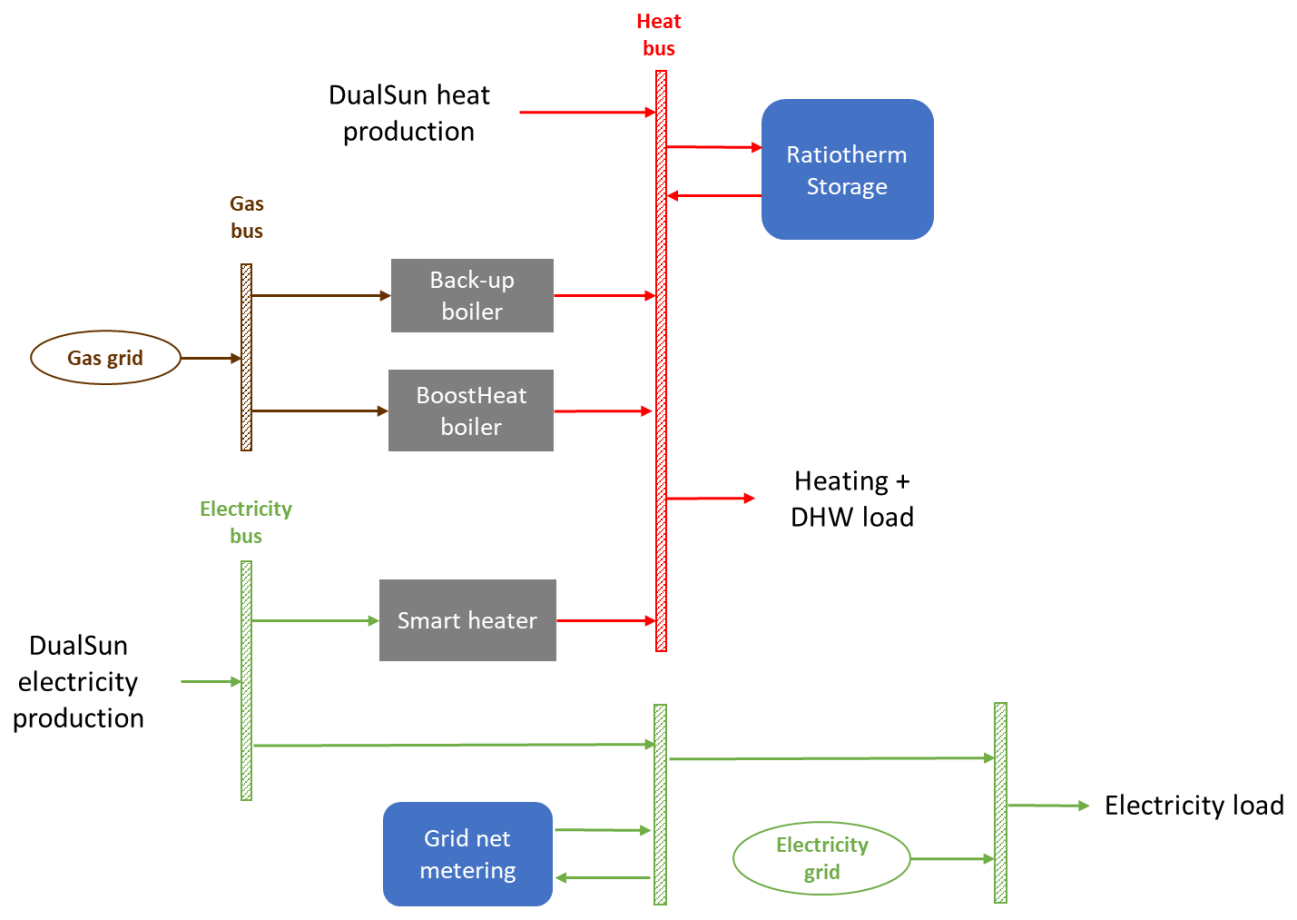
MILP model architecture as implemented in the modelling software. DHW: domestic hot water; MILP: mixed integer linear programming.

Figure 11 gives an overview of the SunHorizon problem architecture as it was implemented inside PERSEE.

The MILP model is based on the following component types:

- Buses: each bus performs a balance of energy flux of its specific energy carrier.
$$\;\sum_{i}P_{in_{i}}^{t}\cdot dt=\sum_{j}P_{out_{j}}^{t}\cdot dt\;(3)$$
  With, for each time *t*, $P_{in_{i}}^{t}$ the i input power to the bus and $P_{out_{j}}^{t}$ the j output powers from the bus
- Energy converters: Smart Heater, BoostHeat heat pump and back-up boiler are energy converters, converting one type of energy carrier to the other with a fixed efficiency.
$$\;P_{out}^{t}=P_{in}^{t}\cdot \eta_{converter}\;(4)$$
  With, for each time *t*, $P_{out}^{t}$ the produced output power of the converter, $P_{in}^{t}$ the input power for the converter and *η _converter_* the efficiency of the converter.
- Storages: Ratiotherm Storage and Grid Net Metering are energy storages that charge and discharge energy to the buses.
$$\;\frac{E_{stored}^{t}-E_{stored}^{t-1}}{dt}=P_{charge}^{t}-P_{discharge}^{t}-K_{loss}\cdot E_{stored}^{t}\;(5)$$
  With, for each time *t*, $E_{stored}^{t}$ the energy stored in the storage, $P_{charge}^{t}$ the charging power of the storage, $P_{discharge}^{t}$ the discharge power of the storage and *K _loss_* an aggregated loss coefficient of the storage.
- Loads and productions: loads and production are imposed time series on a bus.
- Grids: grids offers an infinite source of energy that can be purchased by the system.

In this paper, three objectives functions are compared, the first one on costs minimization in Equation (6) (referred to as MPC on costs), and the second one on GHG emissions minimization in Equation (7) (referred to as MPC on GHG). The last objective function is designed to maximize both self-consumption of the building (which is 100% renewable) and renewable energy use, and is detailed in Equation (8) (referred to as MPC on SELF). This is done by minimizing the gas consumption as well as the non-renewable part of electricity bought from the grid.

$$\;f_{obj_{costs}}=\sum_{t}\begin{array}{l} P_{grid}^{t}\cdot Grid_{price}^{t}\cdot dt+Gas_{consumption}^{t}\cdot Gas_{price}\\ \;\;\;\;\;\;+P_{NetMetering}^{t}\cdot NetMetering_{price}\cdot dt \end{array}\;(6)$$

$$\;f_{obj_{GHG}}=\sum_{t}P_{grid}^{t}\cdot Grid_{CO2intensity}^{t}\cdot dt+Gas_{consumption}^{t}\cdot Gas_{CO2intensity}\;(7)$$

$$\;f_{obj_{MPC}}=\sum_{t}P_{grid}^{t}\cdot Grid_{FFshare}^{t}\cdot dt+Gas_{consumption}^{t}\;(8)$$

With, for each time *t*, $P_{grid}^{t}$ [kW] the power extracted from the electricity grid, $Grid_{price}^{t}$ [€/kWh] the instantaneous electricity price, $Gas_{consumption}^{t}$ [kWh] the instantaneous gas consumption of the heat pump, *Gas _price_* [€kWh] the price of gas in Latvia, $P_{NetMetering}^{t}$ [kW] the power drawn from net metering, *NetMetering _price_* [€/kWh] the fixed fee for grid net metering use, $Grid_{CO2intensity}^{t}$ [gCO2eq/kWh] the instantaneous CO2 intensity of electricity from the grid, *Gas _CO2intensity_* [gCO2eq/kWh] the CO2 intensity of natural gas and $Grid_{FFshare}^{t}$ [%] the instantaneous fossil fuel share of electricity from the grid.

### 2.5 Co-simulation with TRNSYS

As part of the MPC methodology, in order to account for the non-linear phenomenon that cannot be modelled through MILP, feedback from the actual system or detailed simulation model are needed at each timestep to update the state of the system. As the Riga building site with real technology package is not operational yet, co-simulation is implemented with the detailed TRNSYS simulation model that was developed in the early stage of the project on sizing purpose ^10^. Co-simulation is implemented on PEGASE, a platform developed at CEA ^17^ that provides a framework for the design and deployment of advanced control strategies

Co-simulation is usually done through FMU. Even if an open source project for an FMU tool for TRNSYS is available online, compatibility issues between 32 bits TRNSYS model and 64 bits optimization software made the use of standard FMU impossible.

An alternative method was developed in this paper, using a local TCP (Transmission Control Protocol) server and sockets. TCP is a protocol of the Internet Protocol suite, that provides communication services at a lower level than an application program. It relies on a connection between a server and a client. A module was developed in PEGASE to launch a TCP server and a newly developed TRNSYS type is working as the client side.

In actual operation, both models are running in parallel and at the end of each time step, both pause until data through the TCP socket is received.

## 3 Results

In this section, results obtained by the MPC for a yearly simulation for the three objective functions are compared ^18^.

**Figure 12.**
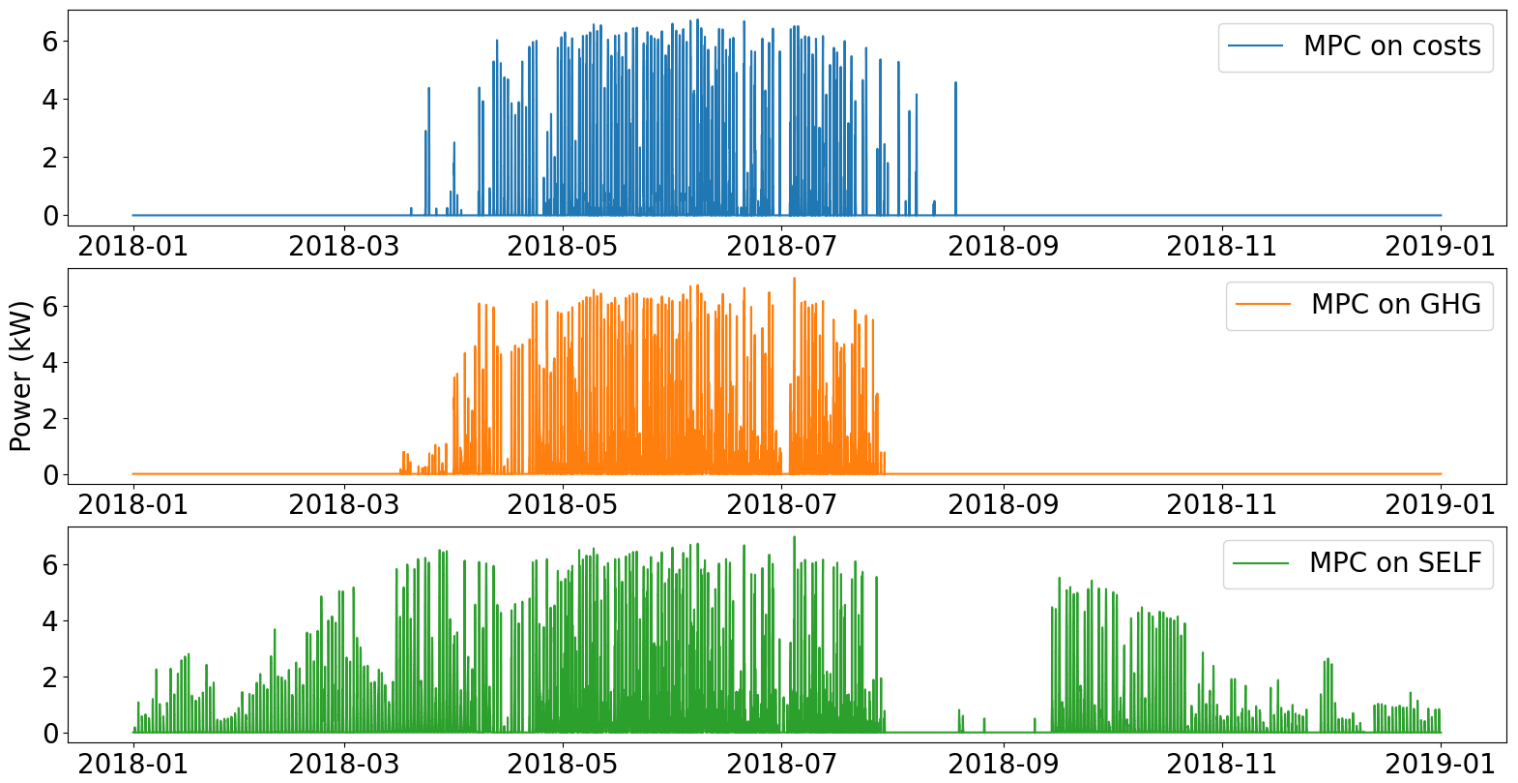
Use of smart heater. GHG: greenhouse gas; MPC: model predictive control; REN: renewable energy use.

Yearly operation of the smart heater is presented on Figure 12.

In the MPC on costs and MPC on GHG scenarios, the operation of the smart heater shows similar trends. The smart heater is mostly used when PV production increases from March to July, In November and December, the control is the same as before March, where the smart heater is not used and heat production is covered with the heat pump only.

For the MPC on SELF, the smart heater has a more predominant role. It is used as soon as PV electricity is produced. This allows for less use of the heat pump and therefore of natural gas. Consumption from the grid is however increased, as it has a higher renewable share than the fossil gas used by the heat pump.

**Figure 13.**
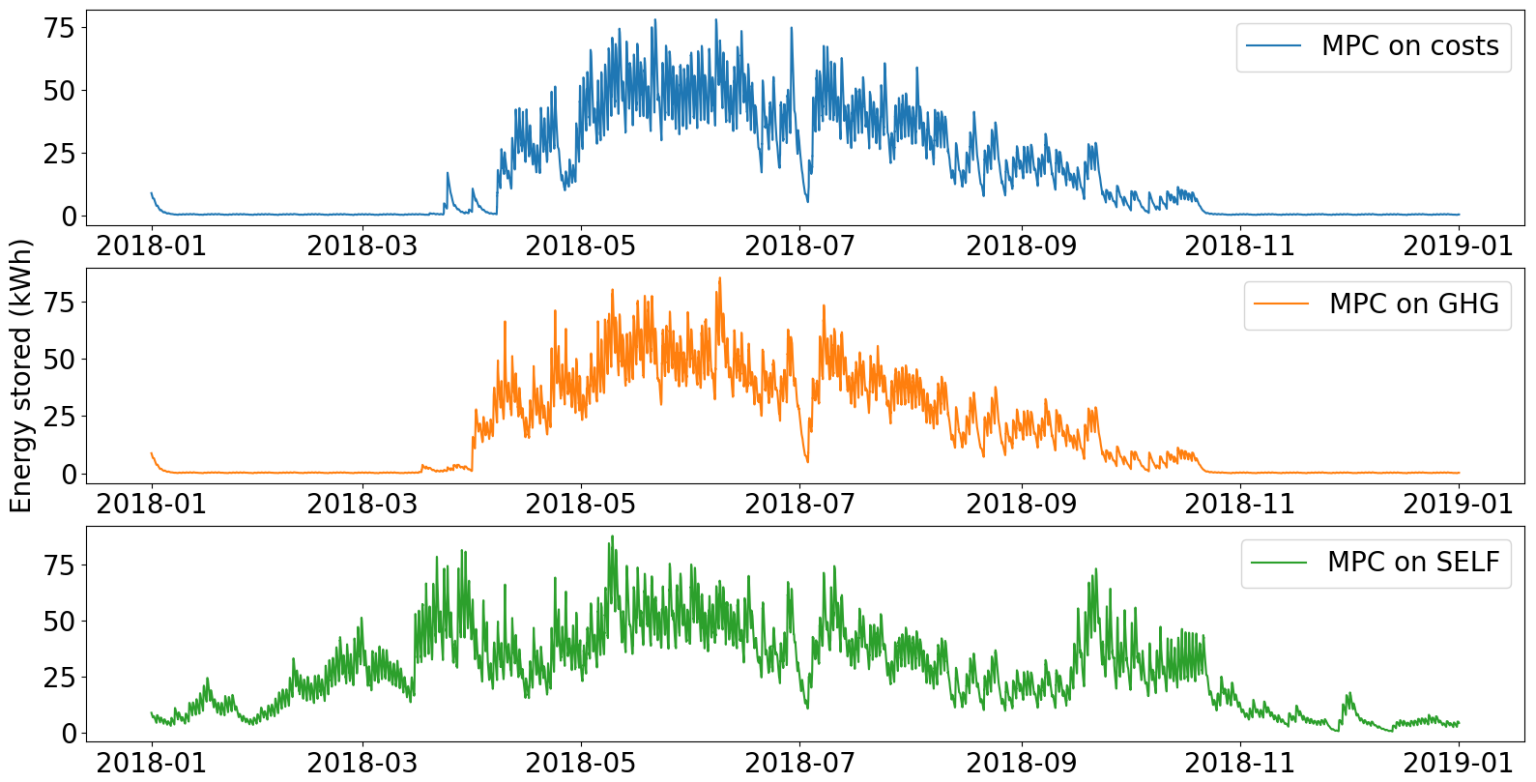
Use of thermal energy storage. GHG: greenhouse gas; MPC: model predictive control; SELF: self-consumption.

**Figure 14.**
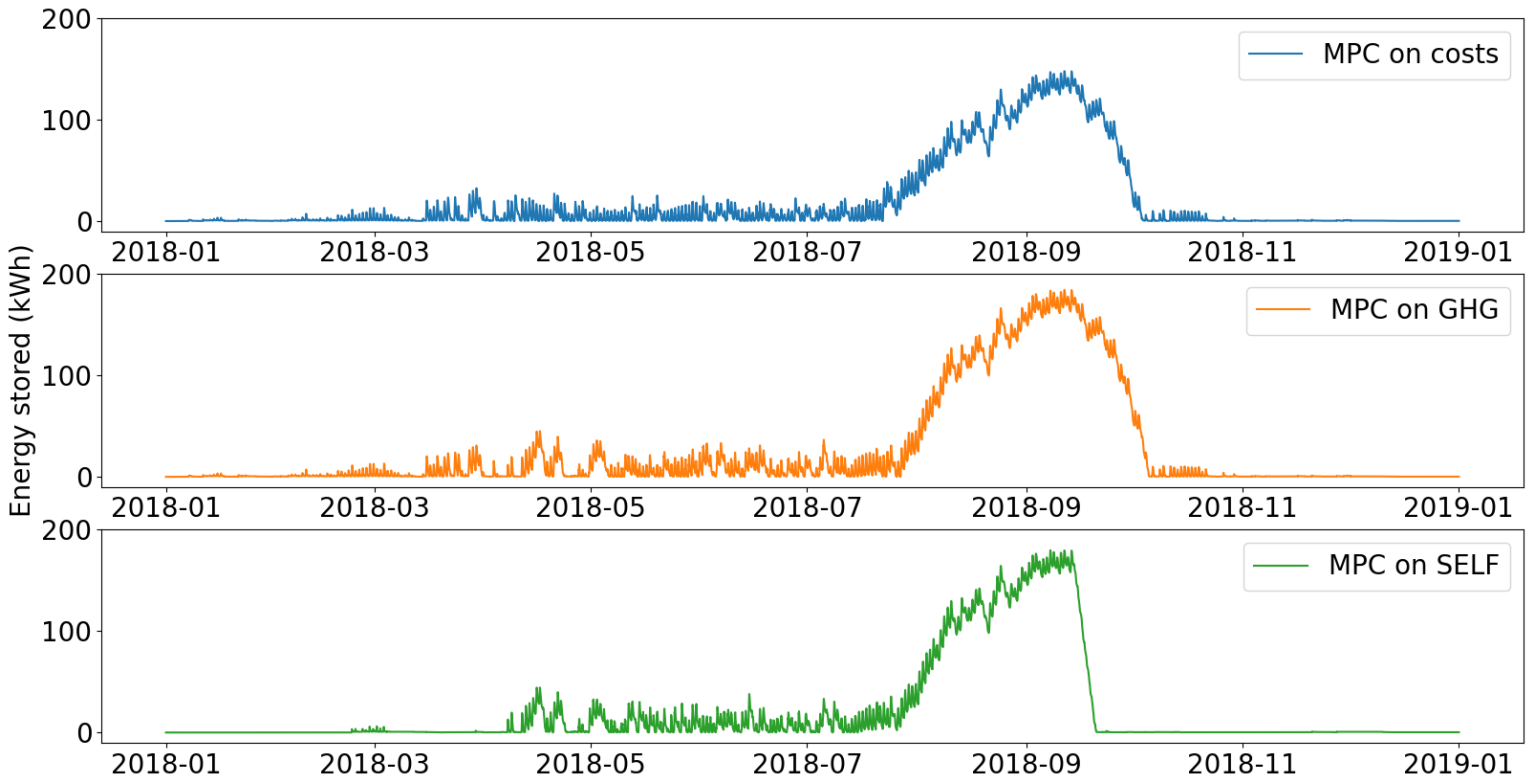
Use of grid metering mechanism. GHG: greenhouse gas; MPC: model predictive control; SELF: self-consumption.

The energy stored in the Ratiotherm storage and the grid net metering are plotted in Figure 13 and Figure 14.

The thermal storage is used in winter on a daily basis but for small amount of energy as the heating demand is high and PVT production low. During summer, the excess of solar thermal production is stored in the water tank, which is use as a buffer before the increase of demand in winter. In summer, the thermal production of PVT panels is indeed higher than the daily heating demand, so despite the heat losses, this production is stored in the tank so it can be used for free at the end of summer when PVT production decreases and heating demand increases.

The main differences between the scenarios lie in the energy stored between November and March. With the MPC on SELF, because of the high use of the smart heater, the thermal energy storage has higher use during this period. Even when heating demand is high, most of the PV production is converted into heat in order to decrease the use of fossil fuel.

Regarding the net metering, for all scenarios electricity is stored mostly at the end of summer, in order to lower the extraction from grid when PV production decreases. However, because more PV production is converted into heat with the MPC on SELF, the cumulative energy stored in net metering is lower. Energy stored through grid metering in SELF scenario drops as soon as the heating demand starts after the summer, so the electricity produced by the PVT panels can be used by the smart heater, in order to minimize the gas consumption.

**Figure 15.**
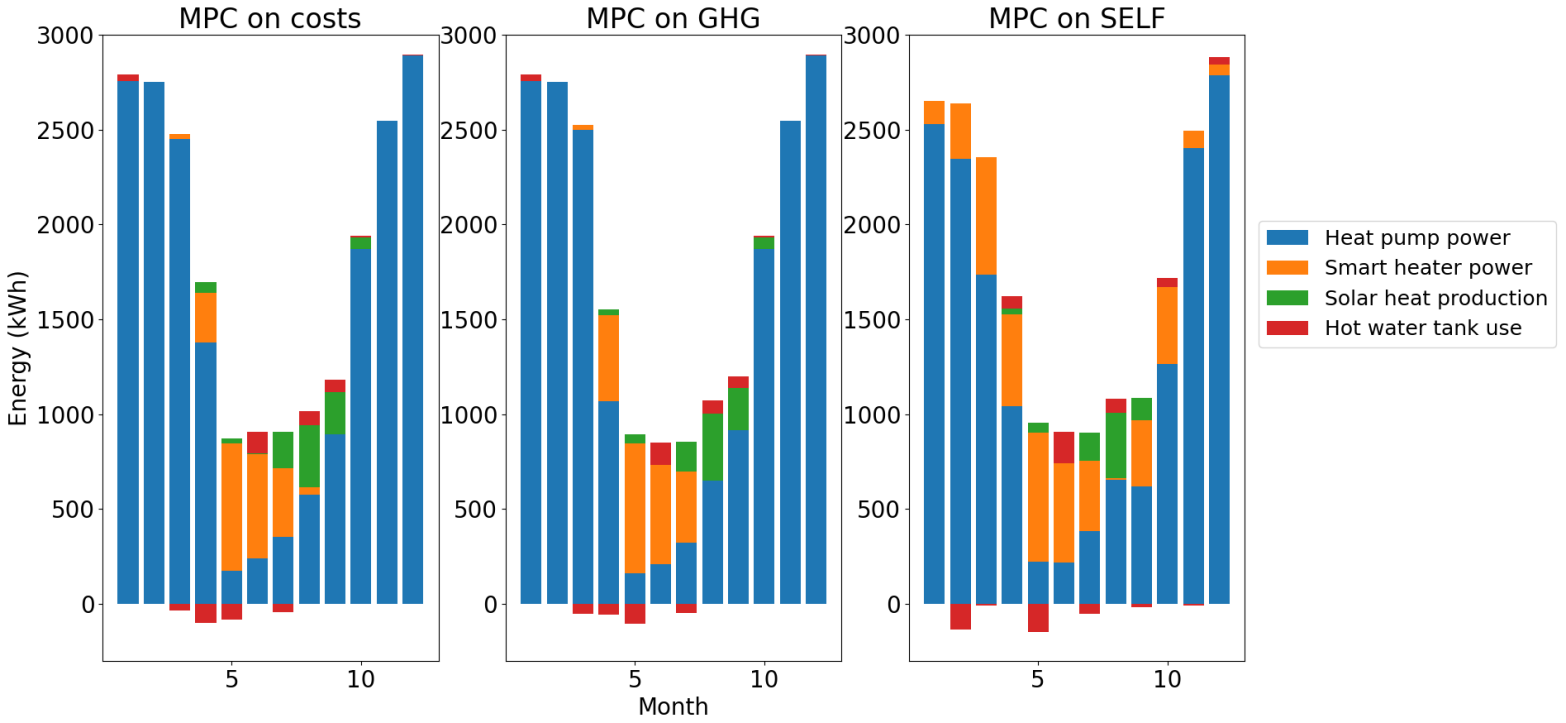
Heat balance per month. GHG: greenhouse gas; MPC: model predictive control; SELF: self-consumption.

**Figure 16.**
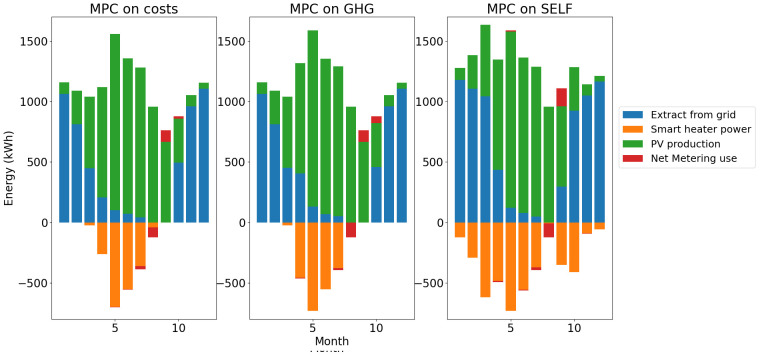
Electricity balance per month. GHG: greenhouse gas; MPC: model predictive control; PV: photovoltaic; SELF: self-consumption.

The total energy balances for heat and electricity are summed up in Figure 15 and Figure 16.

It can be seen in these energy balances that MPC on costs and MPC on GHG have similar behaviors. In all scenarios most of the heat demand is covered by the use of the heat pump. However, as mentioned beforehand, the total heat pump production is lower with the MPC on SELF as the smart heater covers some of the demand outside of summer.

For the electricity balance, the impact of the higher use of smart heater shows a lower use of net metering and higher grid consumption with the MPC on SELF than the two others.

Main indicators for the two control types can be found in Table 3.

**Table 3.** Main indicators for the three control types.

	MPC on costs	MPC on GHG emissions	MPC on SELF consumption
OPEX (€)	1293	1313	1430
GHG emissions (tCO2eq)	6.63	6.59	6.86
Electricity self-consumption (%)	40.3	39.2	46.1
Renewable energy ratio (%)	38.6	39	41.4

GHG: greenhouse gas; MPC: model predictive control; OPEX: operating expenses

The optimization of costs is 1.5% cheaper than the optimization of GHG, and the emission are almost 1% lower in the second scenario. The two first control types offer gains on either costs or GHG emissions differences in the final indicators, however, are small.

This comes from the low level of flexibility of the system, the only control variable being the use of the smart heater. The high CO2 intensity of electricity in Latvia and the low cost of natural gas makes the use of the heat pump an inevitable choice in terms of both costs and GHG emissions.

MPC is however able to profit from the variations in electricity prices or CO2 intensity, to highlight potential gains depending of the chosen control strategy.

The scenario for self-consumption and renewable share results in an increase of both the electricity self-consumption and the renewable energy share. However, this comes with a notable increase in both costs and GHG emissions of the overall system. In this scenario, the smart heater is more used, therefore less gas is bought for heating and more electricity is bought from the grid. As electricity from the Latvian grid has, in average, higher CO2 intensity than natural gas (467 gCO2eq/kWh in average for the grid and 244 gCO2eq/kWh for natural gas), GHG emissions are higher in the scenario. However, as gas is 100% fossil and grid electricity is always partly renewable, the overall renewable share is therefore higher. This control provides interesting results in the case where self-consumption is an important issue, but its economic and environmental interests are low.

## 4 Conclusion

This paper investigates the application of MPC-MILP methodology on an innovative technological package for residential building following possible developments of Latvian context. To proceed with the optimal control, the environmental impacts of the electricity grid are calculated, accounting for imports from neighboring countries. Co-simulation is performed outside of the FMU standard by using TCP protocol. Finally, long-term energy storage is modelled thanks to an optimization problem with variable time step.

Three control strategies were compared in this paper. Optimal control on costs and GHG show that gains can be found within the variations in time series related to the electricity grid, but the overall operation strategies remain similar. Optimal control of renewable share and self-consumption shows another path for control strategy but economic and environmental performances are lower.

These control strategies could be improved by performing internally multi-criteria optimization. Tradeoffs between the three objectives tested in this paper could then be found, but these calculations usually require high computation times. It would also be interesting to test this technological package in other European countries, as lower CO2 intensity in electricity could induce major variations in control between the cost and GHG minimization scenarios. In parallel, further investigations in simulation for such kind of solar and heat pump system are needed to establish the relationships between the size of the thermal storage, its thermal losses and the optimal control’s time horizon. Finally, time series forecasting of weather and electricity related data could be investigated to improve reliability of the results in live implementation.

## Data Availability

Harvard Dataverse: Model Predictive Control of sun-coupled innovative heat pumps: a comparison of economic and environmental optimizations. https://doi.org/10.7910/DVN/3O1RTO ^18^ This project contains the following underlying data: MPC_outputs_costs_scenario.tab MPC_outputs_ghg_scenario.tab MPC_outputs_renshare_scenario.tab Data are available under the terms of the Creative Commons Zero "No rights reserved" data waiver (CC0 1.0 Public domain dedication).

## Software availability

The MPC model was developed on PERSEE and PEGASE, which are proprietary software of CEA. These software programs are used to produce innovative studies for European projects as well as industrial partners. The intellectual property of these programs belongs to CEA, which can sell these studies to companies interested in research and prospective works. Sharing this software cannot be considered as it will deny the added value of CEA in future projects.

Under the ‘obligation to protect results because of legitimate interests or other constraints’ exception of Open Research Europe data policy, we are therefore unable to share the source code associated to this model.

However, in order to replicate the results presented in this article, below is a list of alternative open-source software that can be used as replacements:

- OpenModelica ( https://openmodelica.org/): based on the modeling language Modelica, it offers a free environment to model and analyze complex dynamic systems (alternative to TRNSYS)
- Pyomo ( http://www.pyomo.org/installation) / PulP ( https://pypi.org/project/PuLP/): these Python libraries are used to formulate and solve MILP optimization problems (alternative to PERSEE)
- PyFMI ( https://pypi.org/project/PyFMI/) : this Python library can be used to perform co-simulation with Functional Mock-up Interface standards (alternative to PEGASE)
- Cbc ( https://github.com/coin-or/Cbc) or GLPK ( https://www.gnu.org/software/glpk/) : are free MILP solver (alternative to CPLEX)
