# Supplementary material for: Model predictive control of solar-coupled innovative heat pump: a comparison of economic and environmental optimizations in Latvia
Source: Open Res Eur. 2023 May 17;3:17. Originally published 2023 Jan 25. [Version 2] doi: 10.12688/openreseurope.14992.2 (PMC10921029; doi:10.12688/openreseurope.14992.2)
Supplement: Peer review history

# Reviewer report: Reviewer response for version 2

**DOI:** 10.21956/openreseurope.17260.r38064

**Published:** 2024-03-07

**Authors:** Pengmin Hua

**Affiliations:** 1 Aalto University, Espoo, Finland

In this paper, the authors focused on developing optimal strategies for a residential building with hybrid photovoltaic panels, a gas driven heat pump and a hot water storage tank. They developed a model predictive control method based on mixed integer linear programming optimization, built models in TRNSYS, and implemented the co-simulation on PEGASE. However, the manuscript needs to be majorly modified to further consider the paper for possible indexing.

1. There are many variables and abbreviations in your manuscript, and I suggest you add a Nomenclature to summarise all the variables and abbreviations in your manuscript.
2. There is much related research on applying MPC on buildings with various heating systems. Your manuscript does not accurately introduce the current literature. Thus, you need to add more analysis of related research in **Introduction**.
3. The Conclusion is too general, and you need to describe more details of your research results and emphasize what are your work’s contributions.

Is the study design appropriate and does the work have academic merit?

Yes

Is the work clearly and accurately presented and does it cite the current literature?

Partly

If applicable, is the statistical analysis and its interpretation appropriate?

Yes

Are all the source data underlying the results available to ensure full reproducibility?

Yes

Are the conclusions drawn adequately supported by the results?

Partly

Are sufficient details of methods and analysis provided to allow replication by others?

Yes

Reviewer Expertise:

Model predictive control for buildings with various energy systems

I confirm that I have read this submission and believe that I have an appropriate level of expertise to confirm that it is of an acceptable scientific standard, however I have significant reservations, as outlined above.

# Reviewer report: Reviewer response for version 2

**DOI:** 10.21956/openreseurope.17260.r31738

**Published:** 2023-05-26

**Authors:** Etienne Saloux

**Affiliations:** 1 CanmetENERGY, Natural Resources Canada, Varennes, QC, Canada

Although it is not easy to see the changes in the revised version (no version with track changes), the authors seem to have partially addressed most of the reviewer’s comments overall. The literature review, which has already been slightly extended, could still be further expanded: it only contains a few papers on related topics (2 papers on advanced controls and MPC; 2 papers on building integrated systems) while more initiatives are available in the literature and can be easily found. For the rest, the case study has been described in more depth and technical details have been added to address minor comments (FMI, variable energy price and CO2 intensity forecasting, control variables and time steps, optimization, building load, etc.).

Is the study design appropriate and does the work have academic merit?

Yes

Is the work clearly and accurately presented and does it cite the current literature?

Partly

If applicable, is the statistical analysis and its interpretation appropriate?

Yes

Are all the source data underlying the results available to ensure full reproducibility?

Partly

Are the conclusions drawn adequately supported by the results?

Yes

Are sufficient details of methods and analysis provided to allow replication by others?

Partly

Reviewer Expertise:

Design and advanced controls for buildings and integrated energy systems

I confirm that I have read this submission and believe that I have an appropriate level of expertise to confirm that it is of an acceptable scientific standard.

# Reviewer report: Reviewer response for version 1

**DOI:** 10.21956/openreseurope.16207.r30728

**Published:** 2023-02-09

**Authors:** Daniel Muschick

**Affiliations:** 1 BEST – Bioenergy and Sustainable Technologies GmbH, Graz, Austria

The article presents a simple optimization-based simulation study of a single family home with a thermal-compression, gas fired heat pump, PVT, two thermal storages, an electrical heating element and the possibility to "store" electrical energy in the grid via a specialty of the Latvian net metering billing mechanism. It considers a variable time step prediction horizon to include long-term decisions in the optimization problem and includes a model to represent the time-varying share of renewable energy and CO2 intensity of electricity produced in Latvia or imported from other countries. The main contributions are this time-varying model and the comparison of the results of three optimization tasks: Optimizing costs, optimizing CO2 emissions and optimizing the share of renewables.

 The article currently has multiple **shortcomings**:

- **The title does not match the content**. There is only one heat pump, it is not described at all in the article and its control is not addressed either. It is also not sun-coupled, the heating rod is. Only the second part of the title makes sense. Crucial information is missing both in the title and in the abstract: There is no mention of the Latvian electricity CO2 intensity even though this is - at least in the eyes of the reviewer - one of the most relevant contributions of the article.
- **There is no motivation for the method in the introduction**. The "required developments regarding environmental indicators of the electricity grid", the "alternative method to functional mock-up" and the "modelling of inter-seasonal energy storage" are presented as necessities while never having been mentioned before and not having been motivated (it would, at least, make sense to move the corresponding paragraph one position down to at least have motivation for the environmental indicators).
- **The test case is not sufficiently explained. **There is no information about
  - the efficiency of the PVT modules,
  - on the COP of the heat pump,
  - on the size of the glycol tank (if it is at all relevant),
  - on the temperatures (feed, return) which determine the capacity of the thermal storage,
  - on the loss term describing the storage,
  - on the prices for gas, electricity,
  - on the true workings of the net metering billing mechanism (the use of the word "later" is much too vague
- **The MPC and its interaction with the TRNSYS simulation is not sufficiently explained**
  - If only the smart heater power is a decision variable of the optimizer, where do all the other setpoints come from? How is the heat pump operated? When is energy stored in (one of) the thermal storages? (is it a simple two-point controller with hysteresis based on temperature levels?) When is electricity stored or retrieved from the grid?
  - Is the glycol storage also modeled?
  - What is actually simulated in TRNSYS? Is this simply a 1D water storage model? Is the heat pump simulated?
  - Is perfect foresight assumed? Or are there any forecast algorithms involved?
  - The figure on "Rolling horizon methodology" is misleading ("timeshift" of 1h vs. the smaller hops of... also 1h?)
- **The conclusions seem dubious concerning the difference between GHG and REN objective functions**
  - If the CO2 intensity of the grid in the GHG optimization can be approximated as the CO2 intensity of gas times the fossil fuel share of the network at that time, as would be intuitive, then the two cost functions should be approximately identical. This raises the **question on how the fossil fuel share was calculated** and why it should be so different from the CO2 intensity. Table 2 indicates that MPC on REN share actually *maximizes* GHG emissions, which seems completely counterintuitive.
- **The data provided are the results, not the data required to obtain the results**

These shortcomings must be addressed before the article can be deemed fit for indexing.

**Other comments**:

- Why is the CO2/renewable energy share not considered for power bought back via the net metering process in the cost functions for GHG and REN? It should result in CO2 reduction when sold, and CO2 increase when bought back, thus enabling "CO2 trading".
- The name "seasonal storage" is a bit pretentious and should be re-named to, at most, "long-term storage" (the prediction horizon is 2 months and the storage is actually quite small).
- Figure 11 on the use of thermal storage indicates that the small 1,3 m ^3^ storage is used as a seasonal storage. This is very unlikely given the heat losses this would incur. "Use of thermal energy storage" is also quite misleading, since the storage should of course be "used" in winter, but seems to be empty all the time...?
- Figure 12: Why is there a clean drop in grid metering usage for the REN scenario? This looks weird and requires an explanation. And again: What does "usage" mean? Is this the energy shifted daily, the energy really stored in the grid...
- "Software availability" still only mentions GLPK as an open source alternative to the powerful commercial solvers (CPLEX, Gurobi). In our experience, this solver has been outperformed by at least Cbc and HiGHS for a long time and has not been able to solve many of the optimization problems we posed it. This might have changed, however, the home page has last been updated in 2012. I have the feeling that everybody just copies the same reference over and over again because GLPK pops up almost everywhere in literature.

**Errata**:

- Abstract
  - ... which aims to minimize --> which aim to minimze
- Introduction
  - (HeatRoadMap EU, 2017) --> inconsistent with remaining citations
  - These decisions making .... --> These decision making strategies..
- 2.2 MPC Implementation
  - ... is modelled as a MILP problem, ... --> remove: "problem" (everywhere, not necessary)
  - ... to update ... with actual _information on_ the controlled system
  - ...weather data, heating and electricity demand --> remove space in "dem ands", remove "s"
  - The MPC is using _the_ rolling horizon methodology
  - ... with a 1h time-shift between _them_.
  - ... the behavior of such _a_ storage cannot be forecast
- 2.3 Time series development
  - Figure 5: unit of x axis (hours?) is missing
  - Spot price_s_ for the year 2018 in Lavia _were_ used
- 2.4 System MILP modelling
  - Formula (2) : c should not be a capital letter
  - In general: Try formatting normal text ("costs", "with", "RHS", "LHS") as normal letters, not in italics
  - Formula (8): Index is incorrect: f_{obj_{REN}} instead of f_{obj_{MPC}}
- 2.5 Co-simulation with TRNSYS
  - ..., feedback (no "s" necessary) from _the_ actual system or simulation model _is_ needed ...
  - As _a_ real building site is not operational...
  - ... PEGASE, a platform developed _at_ CEA ...
  - ... compatibility issues between 32 bit (no plural "s" as far as I know) TRNSYS model_s_ and 64 bit optimization software ...
  - ... and at the end of each time step, _both pause_ until data through... (is that what is meant? "it" does not correspond to anything)
- 3 Results
  - Yearly operation of the smart heater and of both (? why both? what about the glycol storage?)
  - ... , as it has a higher renewable share than gas --> what renewable share does gas have in Latvia? Zero as one would expect? --> data is missing
  - The two first control types offer gains on either costs or GHG emissions_;_ differences in the final indicators, however, are _small_.
  - The scenario for renewable share offers an incease _of_ both the electricity... (--> why "offers"? "results in" would be better, I guess)

Is the study design appropriate and does the work have academic merit?

Yes

Is the work clearly and accurately presented and does it cite the current literature?

Partly

If applicable, is the statistical analysis and its interpretation appropriate?

Not applicable

Are all the source data underlying the results available to ensure full reproducibility?

Partly

Are the conclusions drawn adequately supported by the results?

Partly

Are sufficient details of methods and analysis provided to allow replication by others?

No

Reviewer Expertise:

Optimization-based hybrid energy system operation

I confirm that I have read this submission and believe that I have an appropriate level of expertise to confirm that it is of an acceptable scientific standard, however I have significant reservations, as outlined above.

# Response

**Published:** 2023-05-10

**Authors:** Robin Roure

**Affiliations:** Atomic Energy and Alternative Energies Commission, France, France

The authors would like to thank you for your detailed and relevant review. Your comments have been taken into account and allowed us to significantly improve the quality of this article.   All modifications are included in the version 2 that will be published as soon as it is validated by the Editorial Team. Please find below the detailed answer to your review.   We hope it will clarify our work Best regards     Main comments :  

- The title does not match the content.: Explicit mention of the Latvian context and mention of a single heat pump has been fixed in the title. Details have been added in the test case section regarding the description of the whole building energy system which clarified how the PVT collectors’ solar thermal and electricity productions are actually coupled to the hybrid heat pump (consuming gas and electricity) and electric smart heater. In particular, PVT solar heat may flow to the evaporator of the hybrid heat pump through the glycol tank and hybrid heat pump may consume solar electricity produced by PVT panels in parallel to electric smart heater activation.

 

- There is no motivation for the method in the introduction: Additional background and literature review has been added in the introduction to provide clarification on the article motivation

 

- The test case is not sufficiently explained: Additional description of the test case has been added in Section 2.1

 

- The MPC and its interaction with the TRNSYS simulation is not sufficiently explained : Additional description of the interaction with TRNSYS has been added throughout Section 2

 

- The conclusions seem dubious concerning the difference between GHG and REN objective functions: We added the following explanation “In the REN scenario, the smart heater is more used, therefore less gas is bought for heating and more electricity is bought from the grid. As electricity from the Latvian grid has in average CO2 intensity higher than natural gas (467gCO2eq/kWh in average for the grid and 244gCO2eq/kWh for natural gas), GHG emissions are higher in the scenario, but gas is 100% fossil and grid electricity is always partly renewable, therefore the overall renewable share is higher.”

 

- The data provided are the results, not the data required to obtain the results:  This data was required and validated by the editorial team of Open Research Europe. Additionnal data can be obtained on request, however some parts of the models required to fully reproduce the results contain proprietary code.

    Other comments:

- Why is the CO2/renewable energy share not considered for power bought back via the net metering process in the cost functions for GHG and REN? Net metering in the grid was treated as an infinite electricity storage, therefore no CO2 trading with the grid was considered
- The name "seasonal storage" is a bit pretentious and should be re-named [...]: The thermal storage has been renamed “long-term” storage throughout the article according to your comment
- Figure 11 [...] This is very unlikely[...]: We added the following explanation “The thermal storage is used in winter on a daily basis but for small amount of energy as the heating demand is high and PVT production low. In summer, the thermal production from PVT panel is higher than the daily heating demand, so despite the heat losses, this production is stored in the tank so it can be used for free at the end of summer when PVT production decreases and heating demand increases. “ 
- Figure 12: Why is there a clean drop in grid metering usage for the REN scenario? Energy stored through grid metering in SELF scenario drops as soon as the heating demand starts after the summer, so the electricity produced by the PVT panels can be used by the smart heater, in order to minimize the gas consumption. (comment added in the article)
- "Software availability" still only mentions GLPK: At the time of the project, only GLPK was supported by the PERSEE software as an alternative to CPLEX. However Cbc has been recently added to the supported solvers so it has been added to the “Software availability” Section

   Errata:

- All errors were corrected. We thank the reviewer for providing such a careful feedback and apologize for the inconvenience.

# Reviewer report: Reviewer response for version 1

**DOI:** 10.21956/openreseurope.16207.r30727

**Published:** 2023-02-09

**Authors:** Etienne Saloux

**Affiliations:** 1 CanmetENERGY, Natural Resources Canada, Varennes, QC, Canada

The manuscript deals with the evaluation of control strategies targeting different objectives (reduction of energy costs, GHG emissions; increase of renewable energy usage) for a novel system equipped with photovoltaic/thermal panels, gas-driven heat pump, hot water storage tank and smart heater. Results showed that the optimization function slightly impacts the system performance.

 The manuscript is very interesting and looks innovative with the evaluation of different objective functions in a practical context (Latvia). My main comment is that the manuscript is relatively short (less than 5,000 words while the limit is 15,000), the literature review is succinct and some sections should be expanded to provide more details. Detailed comments are given below.

*Technical details: *

- The literature review of similar work in the Introduction section is missing and should be added to report existing work on the same topic, identify research gaps & give more strengths to the contributions, which should also be clearly stated.
- In page 3/16, the authors mentioned that an alternative method to functional mock-up interface standards is required but it is not clear why. More background should be provided.
- In page 4/16, details on the case study are quite succinct. A reference is given for the system sizing but more information should be provided to help the reader better understand the system. For instance, can you explain the principles of the BoostHeat heat pump (absorption heat pump?) since you briefly compare with electrically driven heat pump? Could you provide more details on the smart heater, which seems to store electricity but also converts electricity into thermal energy?
- Section 2.2 discusses MPC implementation and mentions that MILP has been chosen. Could you provide more information about the system modelling and the assumptions that make linear models suitable for such a case study?
- The MPC strategy relies on weather data, variable energy price, and CO2 intensity. Can this information be easily forecasted? A paragraph should discuss this aspect, especially the CO2 intensity, with practical implementation considerations in mind.
- Why has the strategy only considered the smart heater power as the main control variable? Other variables could be used (flow rate in PVT panels, Ratiotherm charging/discharging flow rates, etc).
- Section 2.2 provides information on forecast horizon, but it is a bit confusing. Since the authors highlighted it as a contribution, could you summarize the information in a table and explicitly mention  forecast horizon, control horizon and model time-step?
- In Figure 4, it is not clear what you meant by “cycle” and why there are 21 “arrows”.
- In page 6/16, the sentence starting with "With a typical 48h horizon" is not clear. Why cannot it be forecasted in the short-term while a longer horizon is required?
- The authors discussed the issue of optimization of both short-term and long-term decisions, and the implementation of a new methodology. Previous work has been done on this topic and should be acknowledged. An example I found relevant could be found here ^1^, (no obligation to cite this paper if you do not think it is relevant).
- The authors mentioned that load profiles were calculated but there are no details on it. If it has been part of another study, a reference should be given to provide more confidence on the calculated load.
- In Figure 8, CO2 intensity and REN share have been shown. Could you provide also dynamic energy costs?
- Can you provide a reference for PERSEE software?
- Why is gas consumption included in Eq 8?
- In the Results section (page 11/16), could you discuss why the smart heater is more used in the REN, but not in the GHG scenario?
- The results section shows very interesting results. The authors have mentioned that the design has already been investigated but how the control strategy could impact system design? This aspect could be discussed or further investigated. An example tackling this topic can be found here ^2^, (no need to cite this paper if it is not relevant).

*Format: *

- In Figure 2, could you identify the outdoor unit?
- Page 5/16: demands, not dem ands
- Page 6/16, first paragraph after Figure 4: cannot be forecasted, not forecast
- Page 6/16: air conditioner, rather than chiller
- In Figure 5, consider reformulating “hour of the week” (“hour”?)
- In Figure 12, should the y axis be net metering instead?

Is the study design appropriate and does the work have academic merit?

Yes

Is the work clearly and accurately presented and does it cite the current literature?

Partly

If applicable, is the statistical analysis and its interpretation appropriate?

Yes

Are all the source data underlying the results available to ensure full reproducibility?

Partly

Are the conclusions drawn adequately supported by the results?

Yes

Are sufficient details of methods and analysis provided to allow replication by others?

Partly

Reviewer Expertise:

Design and advanced controls for buildings and integrated energy systems

I confirm that I have read this submission and believe that I have an appropriate level of expertise to confirm that it is of an acceptable scientific standard, however I have significant reservations, as outlined above.

## References

[1] : Representative days selection for district energy system optimisation: a solar district heating system with seasonal storage. *Applied Energy* .2019;248: 10.1016/j.apenergy.2019.04.030 79-94 10.1016/j.apenergy.2019.04.030

[2] : Sizing and control optimization of thermal energy storage in a solar district heating system. *Energy Reports* .2021;7: 10.1016/j.egyr.2021.08.092 389-400 10.1016/j.egyr.2021.08.092

# Response

**Published:** 2023-05-10

**Authors:** Robin Roure

**Affiliations:** Atomic Energy and Alternative Energies Commission, France, France

The authors would like to thank you for your detailed and relevant review. Your comments have been taken into account and allowed us to significantly improve the quality of this article.   All modifications are included in the version 2 that will be published as soon as it is validated by the Editorial Team. Please find below the detailed answer to your review.   We hope it will clarify our work Best regards   Detailed answer :

- The literature review […] is missing:  Literature review has been improved in the introduction
- In page 3/16, alternative method to FMU [...]: More background regarding FMU alternative has been added in the introduction part
- In page 4/16, details on the case study [...]: More details on the case study has been added in section 2.1
- Section 2.2 [...] Could you provide more information about the system modelling ? [...] We added the following explanation “MILP was chosen for the optimization as it provides computation times that are suitable for future live implementation on demosite.”  
- The MPC strategy relies on [...] . Can this this information be easily forecasted? [...]: A dedicated paragraph has been added at the end of Section 2.3
- Why has the strategy only considered the smart heater power as the main control variable?: We added the following explanation “Due to technical constraints of the demosite, only the smart heater in the technological package can be controlled by external control algorithms, therefore it represents our main control variable. Other variables could have been implemented in simulation but it was not representative of the actual controlled system” 
- Section 2.2 provides information on forecast horizon, but it is a bit confusing: Figure 5 has been added to clarify MPC horizon in the model
- In Figure 4, it is not clear what you meant by “cycle” and why there are 21 “arrows”: In Figure 4, “cycle” has been changed to “optimization” for the sake of clarity. The number of 21 arrows is irrelevant, as this figure is only here to explain the concept of rolling horizon (the actual horizon size used in the model is presented in Figure 5)
- In page 6/16, the sentence starting with "With a typical 48h horizon" is not clear [...]: We added “With a typical 48h horizon, the behavior of such a storage cannot be forecast, as electricity can be stored in the grid for more than a few days and used later”
- The authors discussed the issue of optimization of both short-term and long-term decisions [...] . Previous work has been done on this topic and should be acknowledged [...]: TODO : included in literature review
- The authors mentioned that load profiles were calculated but there are no details on it [...]: Reference for load profile calculation has been added
- In Figure 8, CO2 intensity and REN share have been shown. Could you provide also dynamic energy costs?  Figure 7 has been added to show dynamic electricity costs
- Can you provide a reference for PERSEE software?  Reference for PERSEE software has been added
- Why is gas consumption included in Eq 8?  A mistake in the name of the objective function was misleading , it has been corrected
- In the Results section (page 11/16), could you discuss why the smart heater is more used in the REN, but not in the GHG scenario?   The smart heater is more used in the third scenario as it reduces the use of natural gas and increases the use of self-produced heat (which is 100% renewable) 
- [...] The authors have mentioned that the design has already been investigated but how the control strategy could impact system design? [...]  Thank you for your comment, indeed it’s worth investigating further the size of thermal storage when operated by optimal control with long term time horizon and one could expect to draw some general conclusions. The conclusion section is now mentionning it in the perpsective of future simulation workplan.

  All errors were corrected. We thank the reviewer for providing such a careful feedback and apologize for the inconvenience.
